# Nutritional diversity and geographic structure in 45 *Lagenaria siceraria* accessions from southwestern Nigeria, with comparative positioning among related cucurbit seeds

**DOI:** 10.1186/s12870-026-09540-4

**Published:** 2026-07-17

**Authors:** Yemisi O. Olagunju, Adeniyi A. Jayeola, Odunayo J. Olawuyi, Usifo Goodness Adebo, Titilope M. Fasuan

**Affiliations:** 1 Biotechnology Unit, National Horticultural Research Institute, Ibadan, Nigeria; 2 Department of Botany, University of Ibadan, Ibadan, Nigeria; 3 Centre for Research and Development, Avecbrio Consult, Armidale, NSW 2350 Australia; 4 Product Development Program, National Horticultural Research Institute, Ibadan, Nigeria

**Keywords:** *Lagenaria siceraria*, Bottle gourd, Orphan crop, Nutritional variability, Germplasm for biofortification breeding, Antioxidant capacity, Cucurbit germplasm, West Africa

## Abstract

*Lagenaria siceraria* (Molina) Standl. (bottle gourd) is an orphan cucurbit whose seeds are a culturally accepted but scientifically undercharacterised food resource in West Africa, and most existing studies focus on individual accessions or a narrow set of compositional parameters. We therefore characterised the nutritional and phytochemical diversity of 45 accessions collected across four states of southwestern Nigeria (Oyo, Ogun, Osun, Ondo) and grown at the National Horticultural Research Institute, Ibadan, across two seasons under a randomised complete block design with three field replications. Seeds were analysed for proximate composition, mineral content by atomic absorption spectrophotometry, flame photometry and vanadomolybdate spectrophotometry, and antioxidant properties using Folin–Ciocalteu, aluminium chloride colorimetric, phosphomolybdenum and DPPH assays, and data were compared with peer-reviewed values for Citrullus colocynth is, Citrullus lanatus, Cucumis melo and Cucumeropsis mannii. Crude protein varied least (CV 10.78%, range 24.31–38.60%) while total antioxidant capacity varied most (CV 117.30%, range 0.16–7.88 mg TE/g). Phosphorus dominated the mineral profile (mean 711.09 mg/100 g), and a tightly co-varying mineral block (P–K–Ca–Fe, pairwise r 0.80–0.99) was positively associated with antioxidant capacity. A two-factor ANOVA confirmed that all sixteen traits were highly significantly differentiated among accessions (*p* < 10⁻⁵⁵ for every trait) and that neither Year nor Accession × Year was significant for any trait. Principal component analysis explained 59.4% of the variance on PC1 + PC2, and Ward’s D2 clustering resolved three functional groups of fifteen accessions each: a mineral- and antioxidant-rich cluster (C1), a tannin- and carbohydrate-rich cluster (C2) and a protein- and fat-dense cluster (C3). Cluster membership cut across all four states (PERMANOVA F = 0.97, R² = 0.066, *p* = 0.475), indicating accession-level rather than geographic structure. A 100 g serving from C1 accessions (e.g. OS010, OS008, OG008) delivered approximately 120% of the daily phosphorus reference, around 63% of protein and 17–20% of calcium against FAO/WHO adult reference values, although these compositional contributions should be interpreted with phytate- and tannin-mediated bioavailability in mind. *L. siceraria* therefore offers a competitive nutritional profile relative to related cucurbits and a defined germplasm pool for biofortification breeding.

**Supplementary Information:**

The online version contains supplementary material available at https://doi.org/10.1186/s12870-026-09540-4.

## Background

Food insecurity and malnutrition remain significant challenges in sub-Saharan Africa where a large variety of edible plants is available, but this diversity in plants does not prevent the mass of people from getting micronutrient deficiencies, protein malnutrition, and lack of energy as well [1]. Apart from population increase and changing climate, the over-reliance on few staple foods is one factor that not only limits the variety of diet but also causes the lack of enough nutrition. Against this background, the value of neglected and orphan crops is increasingly being realised as a method of changing food systems, increasing nutritional security and supporting agricultural practices that can withstand climate change [2].

In 2022, Vázquez-Martin and Aguilar-Rivera noted that about 369, 000 plant species have been described or identified worldwide, 7, 039 of which are edible plant species; however, only 417 (5.9%) of the edible plant species are listed as major food crops by FAO [3]. The shift to modern agriculture and replacement of traditional cultivars with a few high-yielding varieties have caused the disappearance of about 75% of crop diversity [4]. Today, only nine species account for about two-thirds of the global crop production [3]. The consequences for nutrition, ecosystem function and the resilience of food systems are substantial. International and national research agencies have therefore begun to prioritise dietary diversification, mainstream locally adapted crops and assess orphan species with established nutritional potential [5, 6]. Within the cucurbit family, *Lagenaria siceraria* (Molina) Standl., commonly known as bottle gourd, is one such orphan crop of considerable but underexploited value [7].

**Fig. 1:**
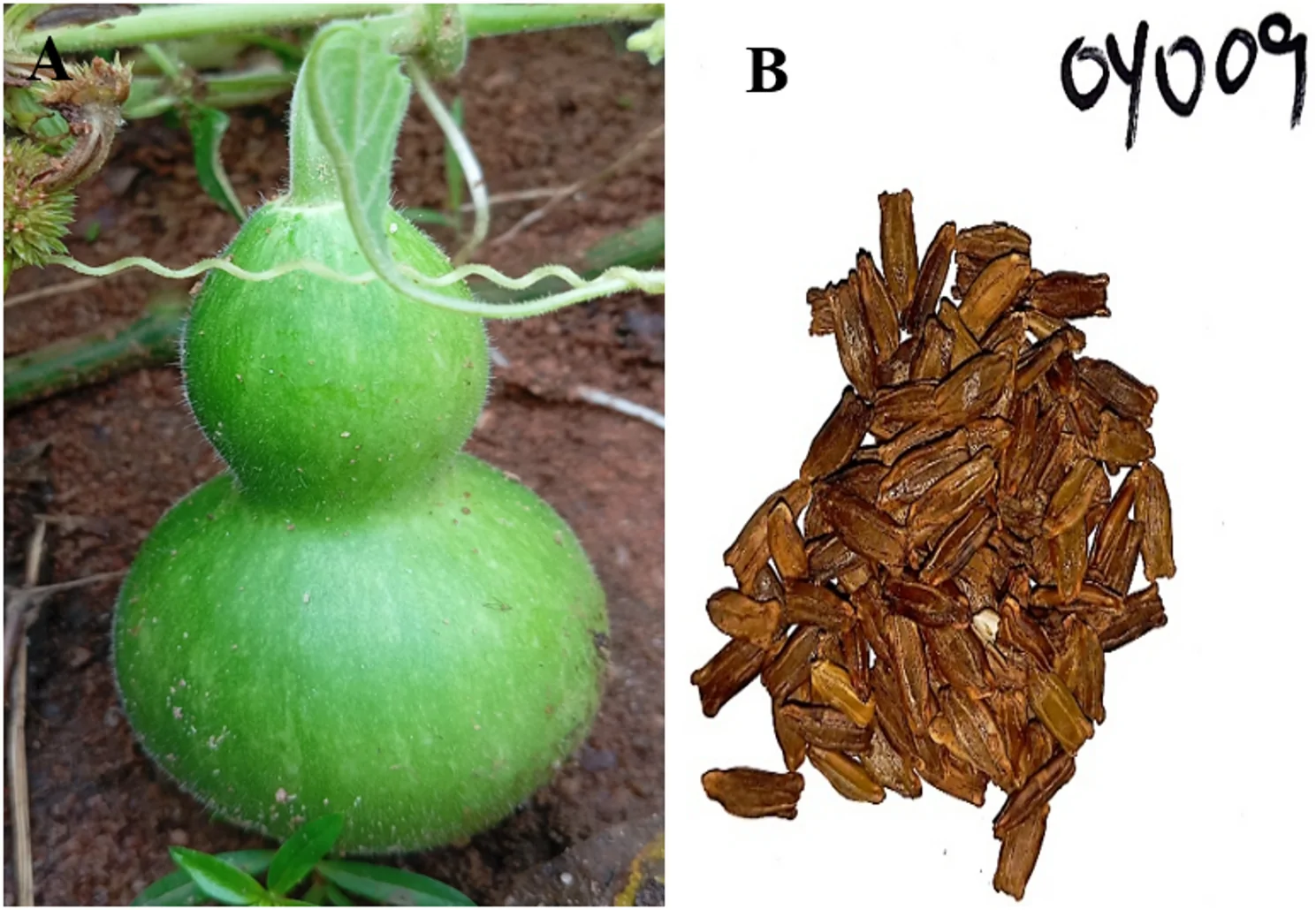
Fruit and seed morphology of *Lagenaria siceraria*: (**A**) mature fruit; (**B**) brown lobed seeds. Source: personal collection

*L. siceraria* is recognised by its large palmate leaves, white nocturnal flowers and hard-shelled fruits containing flattened seeds (Fig. 1). Although it is cultivated mainly for its immature fruits as a vegetable across tropical and subtropical regions, the seeds are themselves an important and often overlooked food resource, particularly in sub-Saharan Africa and parts of Asia. In Nigeria and other West African countries, bottle gourd is a popular crop among smallholder farmers who grow it in different agro-ecological zones. These farmers have their own local varieties of the gourd spread all over the region [8]. Seeds are an excellent source of protein, essential fatty acids and minerals for people since they can be eaten raw, roasted or made into traditional meals. In addition to their nutritional benefits, bottle gourd seeds are also used as powder and ointment in ethnomedicine to treat diabetes, hypertension and liver diseases [9].

Despite this cultural acceptance and the steady availability of seeds as a by-product of vegetable production, *L. siceraria* remains scientifically undercharacterised. Most existing studies have focused on individual accessions or a narrow set of compositional parameters, so the extent of nutritional and phytochemical diversity within the species remains poorly defined. Closely related cucurbit seeds, including those of *Citrullus colocynthis* (L.) Schrad. (egusi melon), *Cucumis melo* (musk/sweet melon) and *Cucumeropsis mannii* (white-seed melon), have been used widely as edible seeds in sub-Saharan Africa, yet comparative nutritional assessments across these species remain fragmented. Filling this gap is necessary if *L. siceraria* is to be promoted, selected and improved on an evidence base comparable to that available for major crops. The present study was therefore designed with three objectives.

First, to characterise the variability in proximate composition, mineral content and antioxidant properties across a panel of 45 *L. siceraria* accessions drawn from four states in southwestern Nigeria. Second, to identify the multivariate structure of this variation through correlation, principal component and cluster analyses, and to test for geographic differentiation among the four states of origin. Third, to position *L. siceraria* within the broader nutritional landscape of related cucurbit seeds (*C. colocynthis*,* Citrullus lanatus*,* C. melo* and *C. mannii*) by drawing on peer-reviewed comparator data, and to assess the contribution that 100 g of seed could make to the recommended dietary allowances of adults. Together, these objectives are intended to support the integration of bottle gourd and related orphan cucurbit crops into nutrition-sensitive food systems in sub-Saharan Africa.

## Methods

### Plant materials and field experimental design

Forty-five accessions of *Lagenaria siceraria* (Molina) Standl. were collected from four states in southwestern Nigeria (Oyo, Ogun, Osun and Ondo): Oyo (*n* = 12), Ogun (*n* = 10), Osun (*n* = 12) and Ondo (*n* = 11). Seed was sourced from farmers’ fields. Botanical identity was confirmed at the herbarium of the Department of Botany, University of Ibadan, and voucher specimens were deposited under accession numbers UIH OY73659 (Oyo), UIH OG74857 (Ogun), UIH OS75864 (Osun) and UIH OD73637 (Ondo). Accessions were collected through stratified sampling: smallholder farms cultivating *L. siceraria* were identified in each state through the State Agricultural Development Programmes, and within each state, accessions were sampled across the principal *L. siceraria*-growing local government areas so that no single LGA contributed more than four accessions.

Field experiments were conducted at the National Horticultural Research Institute (NIHORT), Ibadan, over two consecutive growing seasons (March–August 2023 and March–August 2024). Mean daily maximum and minimum temperatures during the experimental period were 28–30 °C and 22–24 °C, respectively, with rainfall typical of the southwestern Nigerian humid-tropical zone. The experiment used a randomised complete block design with three replications. Each plot contained six plants, spaced 1.5 m within rows and 2.0 m between rows. Standard agronomic management was applied uniformly across plots, including manual weeding and routine crop maintenance. The experimental site has a moderately acidic sandy loam Alfisol (Iwo series) with a pH of approximately 5.8. No fertiliser was applied to the experimental plots during either growing season.

### Sample preparation

At maturity, fruits were harvested from all three field replications of each accession. Seeds were extracted, cleaned of pulp and air-dried at room temperature (27 ± 2 °C) to constant weight. For each accession in each season, seeds from all three field replications were combined into a single composite sample to provide sufficient material for the full panel of analyses. Dried seeds were dehulled, ground to a fine powder using a laboratory mill, and sieved through a mesh of ≤ 1 mm. Powdered samples were stored in airtight containers at 4 °C until analysis. All subsequent laboratory determinations were performed in triplicate on the composite sample, giving three technical measurements per accession per season and six technical measurements per accession across the two seasons (*n* = 270 measurements per trait across the 45-accession panel).

### Proximate composition

Moisture, crude protein, crude fat, crude fibre, ash and carbohydrate contents were determined according to AOAC standard methods [10]. All analyses were carried out in triplicate.

#### Moisture content

Five grams of sample were oven-dried at 105 °C for 6 h to constant weight, and the moisture content was calculated as:

$$\%\;\mathrm{Moisture}=\left[\left(\mathrm{W}_\mathrm{1}-\mathrm{W}_2\right)/\;\mathrm{W}_3\right]\times100$$

where W₁ is the initial weight of dish + sample, W₂ is the weight of dish + dried sample and W₃ is the weight of the sample.

#### Ash content

Five grams of the sample were incinerated in a muffle furnace at 550 °C for 4 h, and the ash content was calculated as the weight of residual ash expressed as a percentage of the original sample weight:

$$\%\;\mathrm{Ash}=\left(\text{Weight of ash / Original weight of sample}\right)\times100$$

#### Crude fat

Crude fat was extracted in a Soxhlet apparatus using n-hexane (boiling point 60–68 °C), following Sha’a et al. [11]. The percentage was calculated as the weight of extracted fat divided by the sample weight, multiplied by 100:

$$\begin{aligned}\%\;\text{Crude fat}=&\text{Weight of extracted fat}\\&/\;\text{Weight of sample}\times100\end{aligned}$$

#### Crude protein

Crude protein was determined by the macro-Kjeldahl method [10]. A 0.5 g sample was digested with concentrated H₂SO₄ in the presence of a catalyst, followed by distillation with 40% NaOH and collection of liberated ammonia in 2% boric acid. The distillate was titrated with 0.01 N HCl. Total nitrogen was calculated as:

$$\begin{aligned}\%\;\mathrm{Nitrogen}=&\left(\mathrm{Titre}\times\text{Normality of HCl}\right. \\& \left.\times14\times\mathrm{V}{\_}\mathrm{flask}\times100\right)\\&/\left(\text{Sample weight}\left(\mathrm{mg}\right)\times\mathrm{V}{\_}{\mathrm{aliquot}}\right)\end{aligned}$$

where 14 is the atomic mass of nitrogen, V_flask is the volume of the digestion flask and V_aliquot is the volume of the digest taken for distillation. Crude protein was obtained as:

$$\%\;\text{Crude protein}=\%\;\mathrm{N}\times6.25$$

#### Crude fibre

Two grams of defatted sample were sequentially digested in 1.25% H₂SO₄ and 1.25% NaOH (30 min each), filtered, washed, dried at 100 °C and finally ignited at 600 °C for 30 min [11]. The crude fibre content was the weight loss on ignition expressed as a percentage of the sample weight:

$$\begin{aligned}\%\;\text{Crude fibre}=&\left(\text{Weight of crude fibre}\right. \\& \left. /\text{Weight of sample}\right)\times100\end{aligned}$$

#### Carbohydrate content

Total carbohydrate was estimated by difference:

$$\begin{aligned}\%\;\mathrm{Carbohydrate}=&100-\left(\%\mathrm{Moisture}+\%\mathrm{Ash}\right. \\& \left.+\%\text{Crude fat}+\%\text{Crude protein}\right. \\& \left.+\%\text{Crude fibre}\right)\end{aligned}$$

### Mineral element analysis

Two grams of seed powder were dry-ashed at 550 °C for 5 h, and the ash was dissolved in 10 mL of 1.0 M HCl, filtered and made up to 100 mL with deionised water. Calcium, magnesium, zinc and iron were quantified by atomic absorption spectrophotometry (Buck Scientific 210VGP, USA) using element-specific hollow cathode lamps and matrix-matched standard solutions [10]. Sodium and potassium were determined by flame photometry (Jenway PFP7) using standard curves prepared at 20–100 ppm. Phosphorus was measured spectrophotometrically (Lemfield Spectrulab 23 A) at 430 nm by the vanadomolybdate method against a phosphorus standard curve [10]. Mineral concentrations were calculated as:

$$\begin{aligned}\mathrm{Mineral}&\;\mathrm{concentration}\left(\mathrm{mg}/100\;\mathrm{g}\right)\\&=\left(\mathrm{Concentration}\left(\mathrm{ppm}\right)\right. \\& \left.\times\text{Dilution factor}\times100\right)\\&/\left(\text{Weight of sample}\times1000\right)\end{aligned}$$

For every analytical batch, instruments were calibrated against matrix-matched standard solutions prepared from analytical-grade reagents, a reagent blank was included to correct for background absorbance, and an internal reference sample was analysed alongside the seed extracts to verify run-to-run consistency.

### Antioxidant activity and phytochemical assays

#### Extract preparation

For each accession in each season, 1 g of finely ground composite seed flour was extracted with 10 mL of 80% methanol (sample: solvent ratio 1:10, w/v) by orbital shaking for 24 h at room temperature (25 ± 2 °C). The mixture was filtered through Whatman No. 1 filter paper, and the filtrate was used as the extract for the TPC, TFC, TAC, DPPH and tannin assays. All assays were performed in analytical triplicate on the same extract.

#### Total Phenolic Content (TPC)

TPC was determined by the Folin–Ciocalteu method [12]. One millilitre of seed extract was mixed with 2.5 mL of 10% Folin–Ciocalteu reagent and 2 mL of 7.5% Na₂CO₃, incubated for 30 min at room temperature, and absorbance read at 765 nm against gallic acid standards. Results were expressed as mg gallic acid equivalents (GAE) per gram of dry weight.

#### Total Flavonoid Content (TFC)

TFC was quantified using the aluminium chloride colorimetric method [12]. Twenty microlitres of extract was added to 2% AlCl₃·6 H₂O, diluted to 10 mL, incubated for 10 min, and absorbance read at 440 nm. Results were expressed as mg quercetin equivalents (QE) per gram of dry weight.

#### Total Antioxidant Capacity (TAC)

TAC was determined by the phosphomolybdenum method, in which an aliquot of the methanolic extract was combined with reagent solution (0.6 M H₂SO₄, 28 mM sodium phosphate and 4 mM ammonium molybdate), incubated at 95 °C for 90 min, cooled to room temperature, and absorbance read at 695 nm against Trolox standards. Results were expressed as mg Trolox equivalents (TE) per gram of dry weight.

#### DPPH radical scavenging activity

A 5 µL aliquot of extract was added to 3.995 mL of methanolic DPPH (2.4 mg per 100 mL methanol), incubated for 30 min in the dark, and absorbance read at 515 nm [13]. Trolox was included as a positive control in each batch but was not used to construct a full calibration curve, and DPPH scavenging is therefore reported as percentage of inhibition rather than as Trolox equivalents. The percentage of DPPH scavenged was calculated as:

$$\%\;\text{DPPH scavenged}=\left[\left(\mathrm{A}{\_}\mathrm{B}-\mathrm{A}{\_}\mathrm{A}\right)/\mathrm{A}{\_}\mathrm{B}\right]\times100$$

where A_B is the absorbance of the blank and A_A is the absorbance of the sample.

#### Tannin content

Seed powder was extracted in distilled water for 24 h and filtered. One millilitre of extract was mixed with 1 mL of Folin–Ciocalteu reagent for 5 min, followed by 2 mL of sodium carbonate solution. After 30 min of incubation in the dark, absorbance was read at 725 nm against tannic acid standards (0–0.4 mg/mL). Results were expressed as mg tannic acid equivalents per gram of sample [14].

### Statistical and multivariate analysis

All analyses were carried out in R version 4.x [15]. Descriptive statistics (mean, standard error, range and coefficient of variation) were computed for each trait from the full 270 measurements (45 accessions × 2 seasons × 3 analytical replicates). To formally test whether the observed nutritional variability was driven by accession differences rather than by between-season environmental effects, a two-factor analysis of variance was fitted for every trait with Accession, Year, and Accession × Year as fixed effects and the three analytical replicates of the pooled field sample as the residual error term. Accession-level means pooled across the two seasons were used as input for all subsequent multivariate analyses. Pairwise relationships among traits were examined by Pearson correlation, with the critical value at α = 0.05 for *n* = 45 accessions calculated as |r| ≥ 0.294 and non-significant cells masked in the visualisation.

Multivariate structure was explored by principal component analysis (PCA) on standardised data using the FactoMineR package. To address the possibility that the strongly co-varying mineral block (P, K, Ca, Fe; pairwise |r| 0.90 to 0.99) was dominating the variance structure, a sensitivity PCA was repeated after removing phosphorus, calcium and iron from the trait matrix. Hierarchical agglomerative clustering was performed on Euclidean distances of standardised trait values using Ward’s D2 linkage. Mean silhouette widths were computed for k = 2 to k = 5 to assess cluster separation, and the optimal cluster number was selected by combining silhouette evidence with biological interpretability. Geographic differentiation was tested by permutational multivariate analysis of variance (PERMANOVA, function adonis2 in the vegan package, 9999 permutations) on Euclidean distances of standardised trait values, with state of origin as the grouping factor. Homogeneity of multivariate dispersion was assessed with PERMDISP (function betadisper) to verify that any PERMANOVA result was not confounded by unequal within-group spread.

Contributions to recommended dietary intakes per 100 g of seed flour were calculated against the following adult reference values: 50 g/day protein and 70 g/day fat from the FAO/WHO/UNU report on Protein and Amino Acid Requirements in Human Nutrition [16]; 1000 mg/day calcium and 13.7 mg/day iron from the joint FAO/WHO Vitamin and Mineral Requirements in Human Nutrition [17]; 700 mg/day phosphorus from the IOM Dietary Reference Intakes [18]; 3510 mg/day potassium and 2000 mg/day sodium from the relevant WHO guidelines [19, 20]; and 25 g/day for crude fibre. R packages used were tidyverse, readxl, vegan, cluster, factoextra, FactoMineR, Hmisc, corrplot and RColorBrewer.

### Comparator data for related cucurbit seeds

To position the present results within the broader cucurbit literature, peer-reviewed proximate, mineral and antioxidant data for seeds of *Citrullus colocynthis*, *Citrullus lanatus*, *Cucumis melo* and *Cucumeropsis mannii* were compiled from full-text articles retrieved through Consensus and verified against the original publications. The comparator studies used analytical pipelines broadly comparable to those applied here: AOAC-based proximate analysis, dry-ashing followed by AAS or flame-photometric mineral quantification, and Folin–Ciocalteu and DPPH-based antioxidant assays [21–27]. Where comparator papers reported antioxidant activity using extract solvents other than those used in the present study (methanolic, hydromethanolic, ethyl acetate or aqueous), this is noted alongside the relevant data, since solvent polarity and extract concentration influence the absolute values reported. Where a study reported on *Citrullus lanatus* under West African “egusi” nomenclature that elsewhere refers to *C. colocynthis* or *C. mannii*, this is flagged in the relevant table footnote.

## Results

### Variability in proximate, mineral and antioxidant traits

The two-factor analysis of variance revealed a highly consistent pattern across all sixteen traits (Table S2). Accession effects were significant at *p* < 10⁻⁵⁵ for every trait, with F-values ranging from 23.74 for moisture to 4446.38 for total flavonoid content. In contrast, the Year main effect was non-significant for all sixteen traits (*p* > 0.61 in every case), and the Accession × Year interaction was likewise non-significant for every trait (*p* > 0.56 in every case). The trait architecture observed in this panel is therefore driven by accession-level differences and is stable across the two growing seasons examined, justifying the use of accession-level means pooled across years for the multivariate analyses that follow.

Substantial variation was observed across the 45 *Lagenaria siceraria* accessions for all sixteen traits examined (Table 1), with coefficients of variation ranging from 10.78% for crude protein to 117.30% for total antioxidant capacity. Among the proximate components, crude protein was the most stable trait (mean 31.71 ± 0.21%, range 24.31–38.60%), while crude fat showed greater variability (mean 30.34 ± 0.49%, range 9.87–42.59%) and carbohydrate content varied even more widely (mean 23.94 ± 0.64%, range 4.79–49.54%). The mineral profile was dominated by phosphorus (mean 711.09 ± 7.88 mg/100 g), followed by potassium (542.81 ± 6.40 mg/100 g) and sodium (329.33 ± 4.10 mg/100 g), with calcium and iron at lower concentrations (184.51 ± 1.47 mg/100 g and 1.51 ± 0.02 mg/100 g respectively). Antioxidant traits were the most variable: total antioxidant capacity ranged from 0.16 to 7.88 mg TE/g (CV 117.30%), and total flavonoid content from 0.14 to 4.31 mg QE/g (CV 84.74%). Total phenolic content showed only modest variation (mean 0.36 ± 0.004 mg GAE/g, CV 17.91%), confirming that the very high variability seen in TAC and flavonoid content reflects compositional differences in the bioactive profile rather than total phenolic concentration alone.

**Table 1:** Descriptive statistics of sixteen proximate, mineral and antioxidant traits in 45 *Lagenaria siceraria* accessions from southwestern Nigeria

Trait	*N*	Mean	SE	SD	Min	Max	CV (%)
Ash (%)	270	5.47	0.05	0.86	3.20	8.03	15.78
Calcium (mg/100 g)	270	184.51	1.47	24.15	126.13	242.56	13.09
Carbohydrate (%)	270	23.94	0.64	10.51	4.79	49.54	43.90
Crude fibre (%)	270	3.82	0.03	0.54	2.69	5.12	14.07
DPPH (%)	270	38.14	1.23	20.23	0.49	73.98	53.05
Crude fat (%)	270	30.34	0.49	8.13	9.87	42.59	26.78
Flavonoid (mg QE/g)	270	1.46	0.08	1.24	0.14	4.31	84.74
Iron (mg/100 g)	270	1.51	0.02	0.29	1.00	2.29	19.00
Moisture (%)	270	4.60	0.08	1.25	1.00	10.00	27.13
Phosphorus (mg/100 g)	270	711.09	7.88	129.46	417.69	1033.21	18.21
Potassium (mg/100 g)	270	542.81	6.40	105.16	332.63	826.74	19.37
Crude protein (%)	270	31.71	0.21	3.42	24.31	38.60	10.78
Sodium (mg/100 g)	270	329.33	4.10	67.36	172.00	445.07	20.45
TAC (mg TE/g)	270	1.75	0.12	2.05	0.16	7.88	117.30
Tannin (mg/g)	270	2.45	0.11	1.77	0.70	6.10	72.49
Total phenolic (mg GAE/g)	270	0.36	0.00	0.06	0.24	0.51	17.91

*N* sample size, *SE *standard error, *SD *standard deviation, *CV *coefficient of variation, *TAC *total antioxidant capacity, *QE *quercetin equivalents, *TE *Trolox equivalents, *GAE *gallic acid equivalents

### Trait–trait relationships

**Fig. 2:**
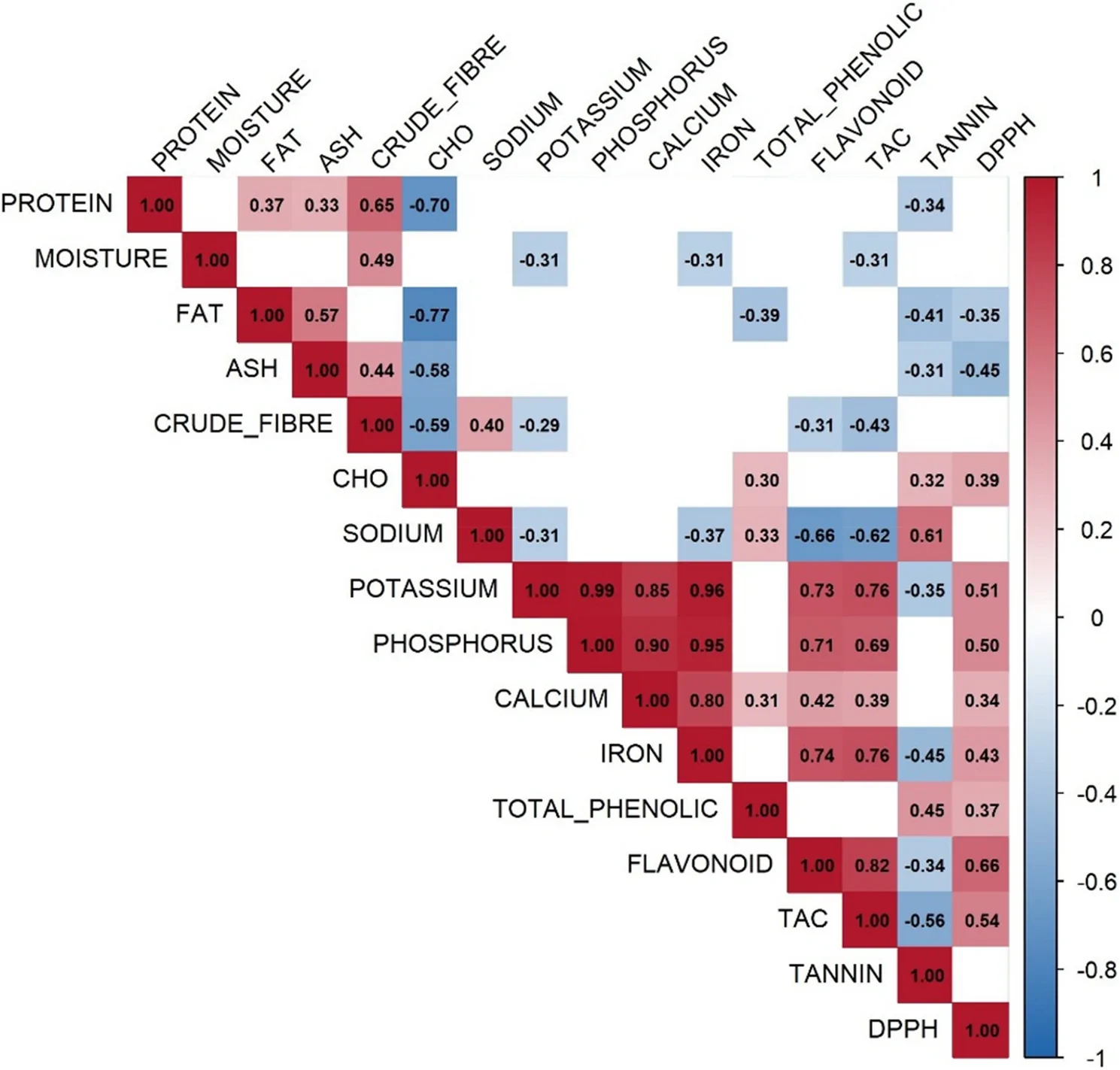
Correlation heatmap among proximate, mineral and antioxidant properties of 45 *Lagenaria siceraria* accessions. Pearson correlation coefficients are colour-coded from − 1 (blue) to + 1 (red)

Pearson correlation analysis revealed three coherent groups of co-varying traits (Fig. 2). The strongest set of positive correlations occurred among the macro- and micro-minerals: calcium, iron, potassium and phosphorus showed correlations between 0.80 and 0.99, with the iron–potassium and potassium–phosphorus pairs reaching 0.96 and 0.99, respectively. This near-collinearity indicates that mineral density in *L. siceraria* seed is a coordinated trait, with accessions tending to accumulate these elements together rather than independently.

Antioxidant traits formed a second, internally consistent cluster. Total flavonoid content and total antioxidant capacity correlated at *r* = 0.82, and both correlated positively with DPPH scavenging (*r* = 0.66 and 0.54, respectively). Notably, the antioxidant block was also positively associated with the mineral block (DPPH–iron 0.76, DPPH–potassium 0.76, TAC–phosphorus 0.71, flavonoid–calcium 0.39), pointing to a co-occurrence of mineral richness and antioxidant capacity within the same accessions.

Several biologically significant negative correlations were observed. Carbohydrate content was inversely related to moisture (*r* = − 0.70), crude fibre (*r* = − 0.77), protein (*r* = − 0.59) and ash (*r* = − 0.57), aligning with its calculation by difference. Tannin demonstrated negative correlations with flavonoid content (*r* = − 0.65) and TAC (*r* = − 0.62), indicating an antagonistic distribution of tannin and other phenolic antioxidants among accessions. Sodium showed negative relations with iron (*r* = − 0.37), flavonoids (*r* = − 0.66) and TAC (*r* = − 0.62), suggesting that accessions with higher sodium levels generally have lower micronutrient and antioxidant contents.

### Multivariate structure and clustering

**Fig. 3:**
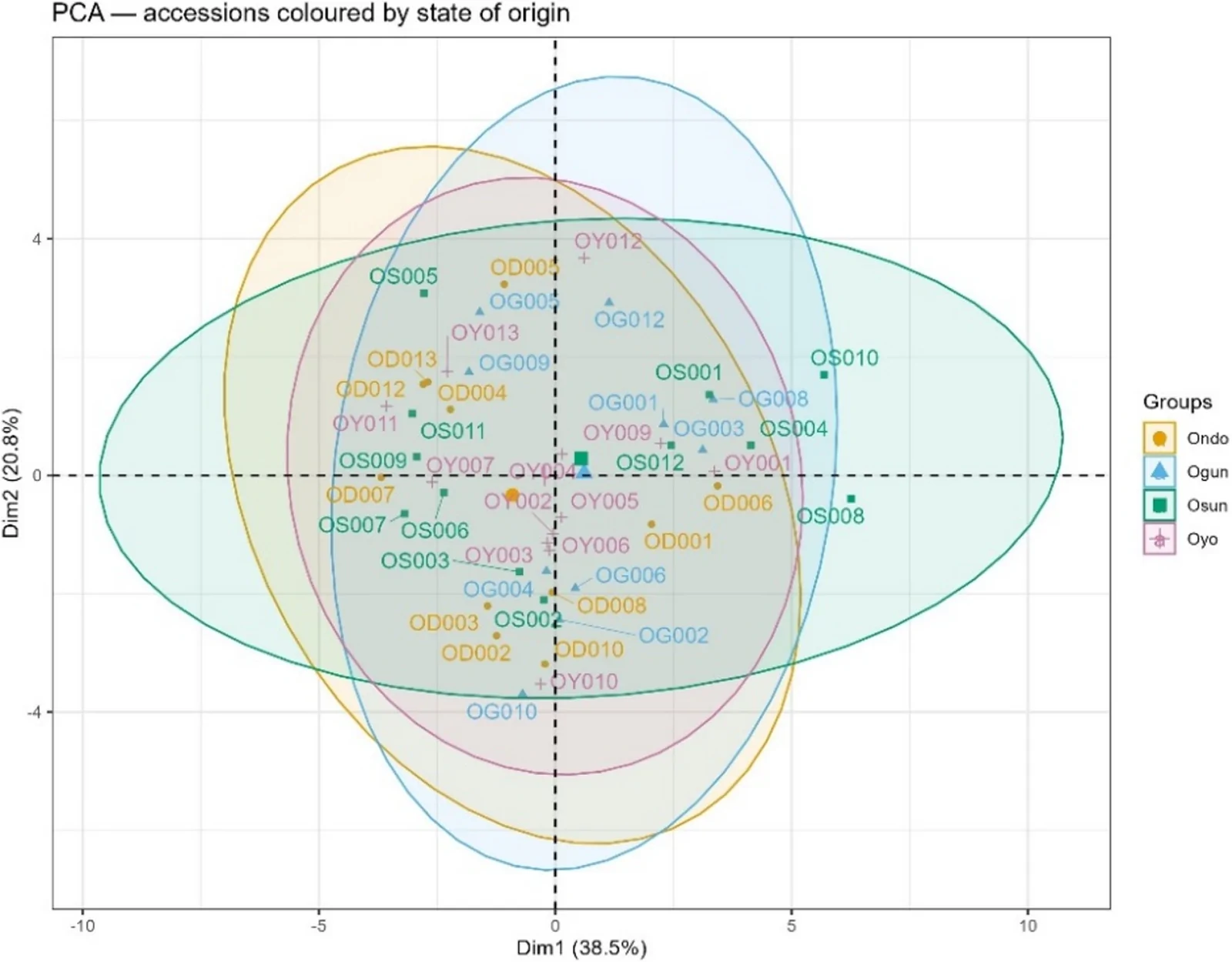
Principal component biplot of 45 *Lagenaria siceraria* accessions coloured by state of origin (Ogun, Ondo, Osun, Oyo). The PCA was performed on the correlation matrix of 16 standardised trait means (pooled across the two seasons and three analytical replicates per accession). PC1 (38.5%) and PC2 (20.8%) jointly explain 59.4% of the total variance. Coloured ellipses are 95% confidence ellipses around the centroid of each state group (statistical, not visual)

Principal component analysis condensed the sixteen traits onto two dominant axes that together explained 59.4% of the total variance (PC1 = 38.5%, PC2 = 20.9%; Fig. 3). PC1 was loaded positively by the mineral and antioxidant traits (iron, phosphorus, potassium, calcium, TAC, flavonoid, DPPH) and negatively by sodium, moisture and crude fibre, separating accessions along a “mineral- and antioxidant-density” axis. PC2 was positively loaded by fat, protein and ash, and negatively by tannin, total phenolic and carbohydrate, distinguishing energy-dense, high-storage accessions from those with carbohydrate- and tannin-dominated profiles. Accessions occupied distinct regions of this PC1–PC2 space, and the projection of accessions coloured by state of origin (Fig. 3) showed substantial overlap among the four states, indicating that the multivariate nutritional signal is not structured by geographic provenance.

**Fig. 4:**
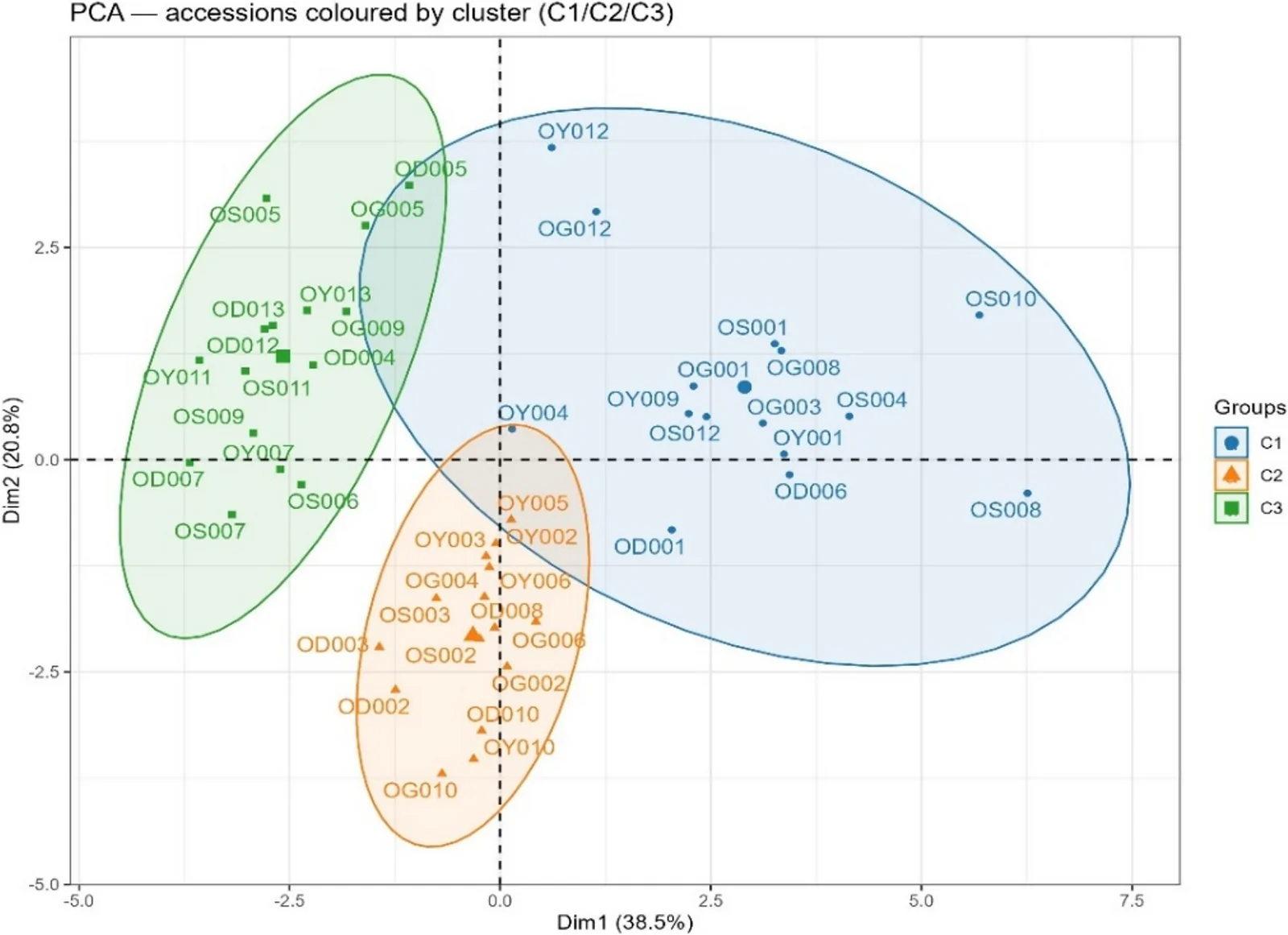
Principal component biplot of 45 *Lagenaria siceraria* accessions coloured by nutritional cluster (C1 = mineral- and antioxidant-rich, C2 = tannin- and carbohydrate-rich, C3 = protein- and fat-dense). Variables included are the same 16 standardised traits as in Fig. 3 (pooled across two seasons and three analytical replicates). Coloured ellipses are 95% confidence ellipses around the centroid of each cluster (statistical, not visual). Cluster assignments were obtained by Ward’s D2 hierarchical clustering on Euclidean distances of the same standardised data matrix

**Fig. 5:**
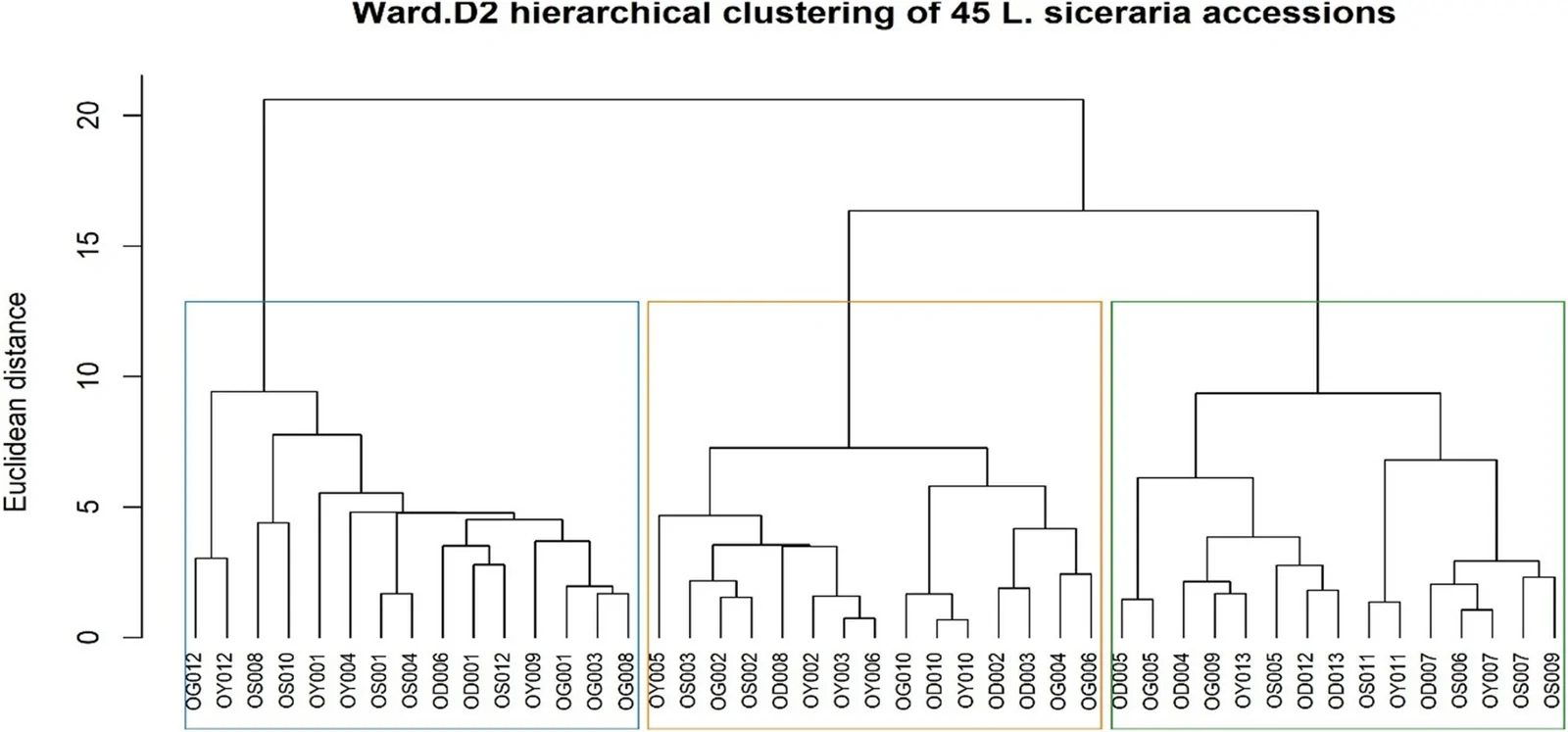
Hierarchical dendrogram of 45 *Lagenaria siceraria* accessions based on Ward’s D2 linkage of Euclidean distances calculated on 16 standardised trait means (pooled across two seasons and three analytical replicates per accession). Coloured boxes indicate the three-cluster partition retained on biological-interpretability grounds (silhouette comparison reported in Additional file 1: Table S3 and Fig. S2). The vertical axis shows Euclidean distance between merged clusters

Hierarchical agglomerative clustering on standardised trait values resolved the 45 accessions into three well-defined groups of 15 accessions each (Figs. 4 and 5; Table 2; cluster-profile boxplots in Additional file 1: Fig. S3). Cluster C1 (*n* = 15) was the mineral- and antioxidant-rich group, with the highest mean values for phosphorus (838.24 ± 22.53 mg/100 g), potassium (656.84 ± 18.80 mg/100 g), calcium (195.77 ± 5.54 mg/100 g), iron (1.83 ± 0.05 mg/100 g), TAC (4.29 ± 0.45 mg TE/g), flavonoid content (2.91 ± 0.25 mg QE/g) and DPPH scavenging (51.23 ± 4.59%). Cluster C2 (*n* = 15) was characterised by the highest tannin (4.87 ± 0.06 mg/g) and the highest carbohydrate content (30.07 ± 2.65%), with intermediate mineral values and modest antioxidant activity (DPPH 46.91 ± 3.20%); this group represents accessions in which a tannin-dominated phenolic profile coexists with a carbohydrate-rich proximate composition. Cluster C3 (*n* = 15) had the highest protein (33.70 ± 0.58%), crude fat (37.35 ± 0.86%), crude fibre (4.06 ± 0.10%) and ash (6.24 ± 0.16%) but the lowest values for mineral density and for every antioxidant trait measured (TAC 0.33 ± 0.02 mg TE/g, flavonoid 0.29 ± 0.02 mg QE/g, DPPH 16.27 ± 1.05%). C3 therefore represents accessions optimised for energy and macronutrient storage at the cost of micronutrient density and bioactive content. The dendrogram (Fig. 5) confirmed C1 as the most divergent group, separating from the remaining clusters at a Euclidean distance of approximately 20, while C2 and C3 separated at a distance of approximately 16.

**Table 2:** Mean trait values for three nutritional clusters identified by hierarchical clustering of 45 *Lagenaria siceraria* accessions across sixteen proximate, mineral and antioxidant traits

Cluster	N	Protein (%)	Moisture (%)	Fat (%)	Ash (%)	Fibre (%)	CHO (%)	Na (mg/100g)	K (mg/100g)	P (mg/100g)
C1	15	31.55 ± 0.94	4.13 ± 0.19	29.20 ± 1.56	5.20 ± 0.20	3.51 ± 0.15	26.97 ± 2.00	259.83 ± 15.58	656.84 ± 18.80	838.24 ± 22.53
C2	15	29.87 ± 0.77	4.88 ± 0.14	24.47 ± 2.14	4.97 ± 0.14	3.90 ± 0.13	30.07 ± 2.65	378.83 ± 8.51	515.33 ± 11.65	692.00 ± 15.43
C3	15	33.70 ± 0.58	4.79 ± 0.45	37.35 ± 0.86	6.24 ± 0.16	4.06 ± 0.10	14.78 ± 1.68	349.33 ± 10.04	456.25 ± 18.63	603.03 ± 28.37

Cluster	Ca (mg/100g)		Fe (mg/100g)	TPC (mg GAE/g)		Flavonoid (mg QE/g)		TAC (mg TE/g)	Tannin (mg/g)	DPPH (%)
C1	195.77 ± 5.54		1.83 ± 0.05	0.35 ± 0.02		2.91 ± 0.25		4.29 ± 0.45	0.82 ± 0.08	51.23 ± 4.59
C2	187.82 ± 4.09		1.39 ± 0.03	0.41 ± 0.01		1.19 ± 0.12		0.63 ± 0.00	4.87 ± 0.06	46.91 ± 3.20
C3	169.93 ± 7.17		1.31 ± 0.05	0.32 ± 0.01		0.29 ± 0.02		0.33 ± 0.02	1.65 ± 0.05	16.27 ± 1.05

*CHO *carbohydrate (by difference), *TPC *total phenolic content, *TAC *total antioxidant capacity. Cluster C1 = mineral- and antioxidant-rich; C2 = tannin- and carbohydrate-rich; C3 = protein- and fat-dense

Mean silhouette widths were 0.285 for k = 2, 0.312 for k = 3, 0.329 for k = 4 and 0.302 for k = 5 (Additional file 1: Table S3; silhouette plot for the k = 3 partition shown in Additional file 1: Fig. S2). Although the silhouette maximum occurred at k = 4, the three-cluster solution was retained because it produced biologically coherent groups aligned with the three end-use targets identified below (mineral- and antioxidant-rich, tannin- and carbohydrate-rich, and protein- and fat-dense). Inspection of the four-cluster solution showed that the additional split occurred within the mineral-rich group without generating a fourth functionally distinct profile, and the three-cluster partition was therefore preferred.

Because the macro- and micro-mineral block displayed pairwise correlations as high as 0.99 between potassium and phosphorus (Fig. 2), a sensitivity PCA was performed after removing phosphorus, calcium and iron from the trait matrix to test whether the multivariate structure was driven by mineral collinearity alone. The reduced PCA explained 59.4% of the total variance on the first two components (PC1 = 35.9%, PC2 = 23.5%), essentially identical to the 59.4% explained by the full PCA (PC1 = 38.5%, PC2 = 20.8%). The three-cluster solution and the relative positions of accessions in the score space were preserved (Additional file 1: Fig. S1), confirming that the multivariate signal reflects coordinated variation across the proximate, mineral and antioxidant trait sets rather than collinearity within the mineral block alone.

### Geographic differentiation

Geographic structure in the nutritional matrix was tested by permutational multivariate analysis of variance (PERMANOVA) on Euclidean distances of standardised trait values, with state of origin as the grouping factor and 9999 permutations. The state effect was not statistically significant (F = 0.97, R² = 0.066, *p* = 0.475), indicating that state of origin accounted for only 6.6% of the total multivariate variance among accessions. PERMDISP returned a non-significant test of dispersion homogeneity (*p* > 0.05), so this result is not confounded by unequal within-group variation.

One-way analysis of variance with state of origin as the fixed factor showed no significant differences among Oyo, Ogun, Osun and Ondo for any of the sixteen traits at α = 0.05 (Table S1). Consistent with this result, the projection of accessions on PC1 and PC2 (Fig. 3) showed extensive overlap among the four states, and each of the three nutritional clusters identified above contained accessions from multiple states. Together, these findings indicate that nutritional and antioxidant variability in the present panel is driven by accession-level differences rather than by geographic provenance, and that diverse nutritional profiles are recoverable from each of the four states sampled.

### Top-performing accessions and dietary contribution

Two Osun accessions stood out as multi-trait leaders (Table 3). OS010 ranked first for phosphorus (1022.10 mg/100 g), potassium (812.12 mg/100 g), calcium (240.20 mg/100 g) and iron (2.24 mg/100 g), and second for DPPH scavenging (71.88%). OS008 ranked second for phosphorus, potassium, calcium and iron, and second for total phenolic content (0.49 mg GAE/g) and TAC (6.67 mg TE/g). Both belong to cluster C1. Protein leaders were OS005 (38.03%), OG012 (37.19%) and OD005 (36.82%), while OG005 (42.09%), OS005 (41.24%) and OG009 (40.58%) led for crude fat; these accessions are predominantly in cluster C3. OY001 (Oyo, C1) recorded the highest TAC (7.72 mg TE/g) and DPPH scavenging (73.04%) in the panel.

**Table 3:** Top three ranking accessions for each proximate, mineral and antioxidant trait among 45 *Lagenaria siceraria* accessions, with corresponding state of origin and measured values

Trait	Rank	Accession ID	State	Value
Protein (%)	1	OS005	Osun	38.03
Protein (%)	2	OG012	Ogun	37.19
Protein (%)	3	OD005	Ondo	36.82
Fat (%)	1	OG005	Ogun	42.09
Fat (%)	2	OS005	Osun	41.24
Fat (%)	3	OG009	Ogun	40.58
Phosphorus (mg/100 g)	1	OS010	Osun	1022.10
Phosphorus (mg/100 g)	2	OS008	Osun	1001.96
Phosphorus (mg/100 g)	3	OG008	Ogun	887.30
Potassium (mg/100 g)	1	OS010	Osun	812.12
Potassium (mg/100 g)	2	OS008	Osun	793.91
Potassium (mg/100 g)	3	OG008	Ogun	700.69
Calcium (mg/100 g)	1	OS010	Osun	240.20
Calcium (mg/100 g)	2	OS008	Osun	234.50
Calcium (mg/100 g)	3	OD005	Ondo	228.20
Iron (mg/100 g)	1	OS010	Osun	2.24
Iron (mg/100 g)	2	OS008	Osun	2.19
Iron (mg/100 g)	3	OG008	Ogun	1.93
Total phenolic (mg GAE/g)	1	OY001	Oyo	0.49
Total phenolic (mg GAE/g)	2	OS008	Osun	0.49
Total phenolic (mg GAE/g)	3	OY005	Oyo	0.45
Flavonoid (mg QE/g)	1	OG012	Ogun	4.31
Flavonoid (mg QE/g)	2	OY012	Oyo	3.50
Flavonoid (mg QE/g)	3	OS008	Osun	3.34
TAC (mg TE/g)	1	OY001	Oyo	7.72
TAC (mg TE/g)	2	OS008	Osun	6.67
TAC (mg TE/g)	3	OY009	Oyo	5.10
DPPH (%)	1	OY001	Oyo	73.04
DPPH (%)	2	OS010	Osun	71.88
DPPH (%)	3	OS012	Osun	70.92

**Fig. 6:**
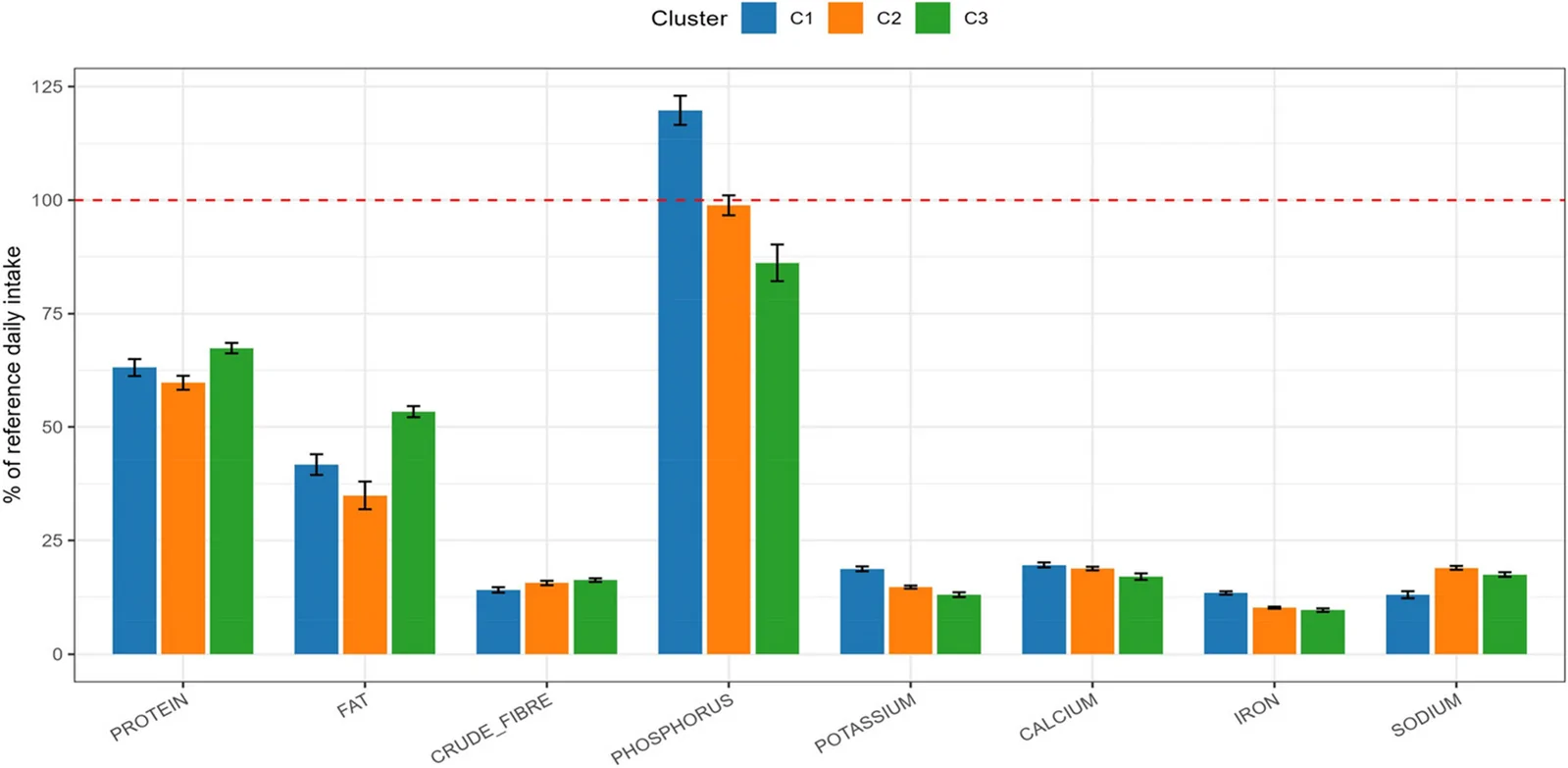
Percentage of the recommended daily allowance (RDA) delivered by 100 g of *Lagenaria siceraria* seed flour across three nutritional clusters. The dashed line indicates the 100% RDA threshold

Based on adult reference intakes, 100 g of *L. siceraria* seed flour delivers substantial proportions of several daily nutrient targets (Fig. 6). Cluster C1 accessions delivered 119.7 ± 3.2% of the daily phosphorus reference, C2 delivered 98.9 ± 2.2% and C3 delivered 86.1 ± 4.1%. Across all clusters, 100 g supplies 59.7–67.4% of the daily protein reference, 35.0–53.4% of fat, 17.0–19.6% of calcium, 13.0–18.7% of potassium, 9.6–13.4% of iron, 13.0–18.9% of sodium and 14.1–16.2% of crude fibre. The mineral-rich C1 accessions, particularly OS010 and OS008, would exceed daily phosphorus requirements from a single 100 g serving and contribute meaningfully to protein, potassium, calcium and iron intake, though bioavailability caveats discussed below should be considered when translating compositional figures into expected nutritional impact.

### Comparative positioning among related cucurbit seeds

*L. siceraria* accessions demonstrated broadly competitive nutritional and antioxidant profiles when compared with four related cucurbit species (Table 4). Mean crude protein (31.71% dw) was comparable to *C. mannii* (33.10–37.45%) and *C. lanatus* (30.63–34.94%), and exceeded lower estimates for *C. colocynthis* (12.06–24.37%) and *C. melo* (27.41–27.80%), though inter-study variability in dehulling status and analytical method limits direct comparison. Mean crude fat (30.34% dw) fell within the broad range documented across all four comparators (8.16–56.61%). Mineral concentrations were notably higher than those of comparator species where data were available. Mean phosphorus (711.09 mg/100 g) far exceeded the only comparable value for *C. colocynthis* (16.8 mg/100 g), mean potassium (542.81 mg/100 g) was substantially greater than *C. lanatus* (236.7 mg/100 g) and *C. melo* (309.1 mg/100 g), and mean calcium (184.51 mg/100 g) surpassed all three comparators (21.4–53.1 mg/100 g). For antioxidant parameters, total phenolic and flavonoid contents were lower than methanol extracts of *C. colocynthis*, though DPPH scavenging activity (38.14%) was within the range reported for *C. mannii* (50.0–51.65%). Pervasive data gaps across comparator species precluded broader antioxidant comparison. Collectively, these findings position *L. siceraria* as a nutritionally competitive cucurbit, particularly with respect to mineral density.

**Table 4:** Comparative proximate, mineral and antioxidant profile of *Lagenaria siceraria* seed and four related cucurbit species

Parameter	L. siceraria (this study)	C. colocynthis	C. lanatus	C. melo	C. mannii	Sources
Crude protein (% dw)	31.71 (24.31–38.60)	12.06; 24.37	30.63; 34.94 ± 0.18	27.41; 27.80	33.10 ± 0.16; 35.46–37.45	[21–23, 28, 29]
Crude fat (% dw)	30.34 (9.87–42.59)	8.16; 56.61	24.50 ± 0.29; 27.40 ± 1.31; 49.05	30.65; 34.60 ± 1.62; 45.20	31.33 ± 0.27; 45.06–49.57	[21–23, 28, 29]
Carbohydrate (% dw)	23.94 (4.79–49.54)	10.88; 24.67	4.52; 24.06 ± 0.08	20.60; 29.96	2.63–3.43; 35.40 ± 0.51	[21–23, 28, 29]
Phosphorus (mg/100 g)	711.09 (417.69–1033.21)	16.8	NR	NR	NR	[21, 23, 30]
Potassium (mg/100 g)	542.81 (332.63–826.74)	5.10	236.7	309.1	NR	[21–23, 30]
Sodium (mg/100 g)	329.33 (172.00–445.07)	NR	98.6	61.5	223.92–246.99	[22, 23, 31]
Calcium (mg/100 g)	184.51 (126.13–242.56)	21.4	30.8	53.1	NR	[22, 23, 30]
Iron (mg/100 g)	1.51 (1.00–2.29)	NR	NR	NR	NR	[23]
Total phenolic (mg GAE/g)	0.36 (0.24–0.51)	60.45 (methanol); 10.02 (HM)	NR	1.50	NR	[22, 32–34]
Total flavonoid (mg QE/g)	1.46 (0.14–4.31)	46.66 (methanol); 6.20 (EA)	NR	0.74	NR	[22, 32–34]
DPPH scavenging (%)	38.14 (0.49–73.98)	73.55 at 50 µg/mL; IC50 274.78 µg/mL	NR	NR	50.0–51.65 at 4 mg/mL	[22, 32–34]
Total antioxidant capacity (mg TE/g)	1.75 (0.16–7.88)	FRAP A0.5: 99.7–119.37 µg/mL	NR	NR	NR	[22, 31, 34]
Tannin content (mg/g)	2.45 (0.70–6.10)	Detected (qualitative only)	29.66	NR	NR	[21, 32]

Values are reported as in the source publications. They are not directly comparable across studies because of differences in species, dehulling status, sample preparation, extraction solvent, analytical method and reporting basis. *L. siceraria* values are mean (range) for *n* = 270 measurements (45 accessions × 2 seasons × 3 analytical replicates). *dw *dry weight, *HM *hydromethanolic, *EA *ethyl acetate, *GAE *gallic acid equivalents, *QE *quercetin equivalents, *TE *Trolox equivalents, *FRAP *ferric reducing antioxidant power, *IC50 *concentration at 50% scavenging

NR, not reported in the retrieved comparator sources or excluded from comparison where reported values fell outside the biologically plausible range for cucurbit seeds. Multiple values separated by semicolons within a single cell are independent reports from different source papers

## Discussion

The 45-accession panel of *Lagenaria siceraria* described here positions the species as a nutritionally competitive cucurbit seed, with a multivariate trait architecture that mirrors patterns documented in better-studied cucurbits and oilseeds. Mahapatra et al. [35] reported significant accession-level variation in mineral and antioxidant traits across 91 Indian *L. siceraria* genotypes and identified positive associations between phenolic content and micronutrient density. The convergence of that finding with the present results indicates that mineral–antioxidant coupling is a species-level property of *L. siceraria* rather than an artefact of any one collection. The protein range (24.31–38.60%) brackets values reported for *Cucumeropsis mannii* by Karaye et al. [21] and Nwoke et al. [23] (Table 4), placing *L. siceraria* among the higher-protein members of the West African cucurbit seed pool. The trait variability spans nearly two orders of magnitude in total antioxidant capacity, a pattern Lee et al. [36] attributed in legumes to differences in phenolic composition rather than total phenolic load.

Crude protein varied least (CV 10.78%; mean 31.71 ± 0.21%; range 24.31–38.60%) and total antioxidant capacity varied most (CV 117.30%; range 0.16–7.88 mg TE/g). The mineral profile was dominated by phosphorus (711.09 ± 7.88 mg/100 g), potassium (542.81 ± 6.40 mg/100 g) and sodium (329.33 ± 4.10 mg/100 g). These ranges fall within the upper range of cucurbit seed values documented by Saeed et al. [22] and Karaye et al. [21], and the wide spread in antioxidant traits points to genotype-level differences in bioactive composition.

Mean crude protein (31.71%) is comparable to *C. lanatus* (34.94%; [21]) and *C. mannii* (35.46–37.45%; [23]), and substantially exceeds *C. colocynthis* (12.06–24.37%; [37, 38]). The protein discrepancy within *C. colocynthis* between those two papers reflects whether seeds were analysed whole or dehulled, and comparator data must therefore separate dehulled from whole-seed values when drawing nutritional inferences. The crude fat range (9.87–42.59%) is wider than that of any single comparator species. The mean phosphorus content (711.09 mg/100 g) exceeds values for *C. lanatus* (450 mg/100 g) and *C. mannii* (511 mg/100 g) reported by Karaye et al. [21], placing *L. siceraria* among the most phosphorus-rich cucurbit seeds in the regional literature. Mean calcium (184.51 mg/100 g) is markedly higher than the 30.8 mg/100 g for *C. lanatus* and 53.1 mg/100 g for *C. melo* reported by Saeed et al. [22]. Mahapatra et al. [35] found strong positive correlations between total phenol content and magnesium, iron and zinc across 91 Indian accessions, providing cross-population support for the mineral–antioxidant association observed here.

Mean total phenolic content (0.36 mg GAE/g) is lower than the 60.45 mg GAE/g reported for methanolic *C. colocynthis* extracts by Abdallah et al. [25], but this gap likely reflects differences in extraction solvent rather than a real species-level deficit; Lee et al. [36] showed that TPC values vary substantially with solvent system even within a single species. Flavonoid content (0.14–4.31 mg QE/g) extends well above the 0.74 mg QE/g reported for *C. melo*, indicating that some accessions merit further phytochemical characterisation.

The negative correlations linking carbohydrate to moisture (*r* = − 0.70), crude fibre (*r* = − 0.77), protein (*r* = − 0.59) and ash (*r* = − 0.57) reflect both the arithmetic of carbohydrate-by-difference calculation and the carbon-partitioning trade-offs documented across oilseeds and legumes. Kambhampati et al. [39] showed that carbon allocated to raffinose-family oligosaccharides during soybean seed development draws from the same pool that feeds protein and lipid synthesis, and Shi et al. [40] proposed that the negative protein–oil correlation is mainly due to competition between carbon and nitrogen during seed-filling. Hossain et al. [41] quantified this trade-off across five Brassicaceae oilseeds and found that a 1-unit increase in seed oil corresponds to a 0.69-unit decrease in protein. Because carbohydrate was calculated by difference, its negative correlations with other components partly reflect a mathematical constraint; the biological interpretation is better supported by the directly measured protein–fat positive correlation (*r* = 0.37).

The mineral co-variation block exhibits the strongest trait association in the dataset. The near-collinear accumulation pattern illustrated by the iron–potassium (*r* = 0.96), potassium–phosphorus (*r* = 0.99) and calcium–phosphorus (*r* = 0.90) correlations is consistent with seed-storage biochemistry as described by Martínez-Ballesta et al. [42], wherein phytic acid forms complexes with potassium, calcium and divalent micronutrient cations, so that reductions in phytate levels in mutant lines lead to simultaneous reductions in all complexed minerals. The Arabidopsis ionome study by Campos et al. [43] and the maize seed ionome work of Roller et al. [44] both confirmed that mineral correlations are largely positive and consistent across environments, making the *L. siceraria* mineral cluster biologically interpretable.

The strong negative correlations between tannin and both flavonoid content (*r* = − 0.56) and TAC (*r* = − 0.56) reflect metabolic competition within the phenylpropanoid pathway. Dixon and Sarnala [45] highlighted how flavan-3-ol monomers can be funnelled either to proanthocyanidin polymers or to soluble flavonoid end-products, regulated by anthocyanidin reductase and dihydroflavonol reductase. Wang et al. [46] demonstrated this trade-off directly: tobacco lines overexpressing the proanthocyanidin pathway showed enhanced proanthocyanidin content alongside decreased anthocyanin pigmentation. Lu et al. [47] confirmed in soybean that distinct transcription-factor sets activate the proanthocyanidin versus the soluble-flavonoid branch, establishing that the partitioning is regulated rather than stochastic.

Total phenolic content (CV 17.91%) and TAC (CV 117.30%) appear decoupled, implying that phenolic variety rather than total amount drives antioxidant variation among accessions. Lee et al. [36] and Majid et al. [48] as also reviewed by Sadowska-Bartosz and Bartosz [49] found that DPPH scavenging was not always concordant with Folin–Ciocalteu TPC among legume and date-seed cultivars, attributing this to differences in phenolic composition rather than total phenolic content. Accessions such as OG012 and OY001, which achieved the highest flavonoid and TAC values respectively, appear differentiated by specific phenolic compositions and therefore deserve targeted phytochemical characterisation. The positive relationships between antioxidant and mineral groups (DPPH–iron *r* = 0.76, DPPH–potassium *r* = 0.76, TAC–phosphorus *r* = 0.71) agree with Mahapatra et al. [35], strengthening the case for a real biological association between micronutrient density and bioactive content in *L. siceraria*, possibly mediated by shared regulatory pathways during seed maturation.

The three nutritional clusters (Table 2; Figs. 4 and 5) describe a clean functional partitioning of the panel. Cluster C1 (*n* = 15) returned the highest means for phosphorus, potassium, calcium, iron, TAC, flavonoid and DPPH scavenging. Cluster C2 (*n* = 15) carried the highest tannin and carbohydrate means with intermediate mineral profiles. Cluster C3 (*n* = 15) showed the highest means for protein, fat, fibre and ash but the lowest mineral and antioxidant values. The dendrogram confirms C1 as the most multivariately distinct group, separating from the remaining clusters by a greater Euclidean distance than the C2–C3 separation. This three-way partitioning maps onto three distinct breeding targets. C1 accessions, exemplified by OS010, OS008 and OG008, are the natural starting material for biofortification programmes targeting mineral density combined with antioxidant capacity; the convergence of top-ranking accessions for phosphorus, potassium and iron within a single unsupervised cluster constitutes internal cross-validation of the multivariate analysis. C3 accessions, exemplified by OS005, OG005 and OG009, constitute the storage-protein and energy-density pool for food-fortification applications. C2 accessions are nutritionally less attractive for direct consumption given their high tannin loading, which would reduce mineral bioavailability through phytate–tannin–mineral complex formation and impart astringency to seed flour.

The use of multivariate clustering for cucurbit germplasm classification has clear precedent. Mitić et al. [50] classified nine edible *Cucurbita* species using PCA and hierarchical cluster analysis on antioxidant profiles. Chithra et al. [51] employed the same approach across 40 sponge gourd genotypes for 32 nutritional and yield traits. Meghashree et al. [52] applied Ward’s clustering to bitter gourd accessions and found four mineral-and-vitamin clusters. Mallikarjuna et al. [53] split 96 bitter gourd genotypes into seven clusters and noted that the first two PCA components accounted for more than 50% of total compositional variation, close to the value obtained here. Didonna et al. [54] similarly used multivariate analysis to profile nine *Cucumis melo* landraces. This methodological convergence supports the analytical framework adopted here. The three-cluster solution is supported by the silhouette-based selection rule and the dendrogram cutting point; the absence of state-of-origin structure within clusters — each containing accessions from multiple states — confirms that the partitioning reflects compositional biology rather than geographic confound.

The strong intra-block mineral correlations (pairwise |r| 0.90–0.99) suggest that potassium, measurable by flame photometry, can serve as a low-cost proxy for the other minerals during early-stage germplasm screening. The high TAC–flavonoid correlation (*r* = 0.82) similarly suggests that TFC by aluminium chloride colorimetry could proxy for the more elaborate phosphomolybdenum TAC assay. For conservation, sampling representative accessions from each cluster rather than each state would more efficiently capture the full nutritional diversity of the panel. Validation of proxy-trait heritability requires field-block-level replication through to the laboratory phase, which the present design cannot provide.

Phosphorus, calcium and iron concentrations are total values and do not equate to bioavailable intake. Phytate was not quantified, and dietary contribution figures should be interpreted as compositional ceilings rather than expected absorbed intake. Cluster C1 has a bioavailability advantage over C2 by virtue of its substantially lower tannin loading (0.82 ± 0.08 mg/g versus 4.87 ± 0.06 mg/g in C2), since tannins form additional ternary complexes with phytate and minerals, strengthening the case for C1 as a biofortification target even before phytate is directly measured. The IOM tolerable upper intake level for phosphorus in adults (4000 mg/day) comfortably exceeds the ~ 840 mg supplied by 100 g of a C1 accession, but routine consumption of larger quantities by individuals with compromised renal function warrants consideration.

The two-factor ANOVA demonstrated that all sixteen traits varied highly significantly among accessions (*p* < 10⁻⁵⁵) but neither Year nor Accession × Year was significant for any trait. A one-way ANOVA with state of origin as the grouping factor showed no significant differences among Oyo, Ogun, Osun and Ondo for any trait (α = 0.05; Table S1), confirmed by PERMANOVA (F = 0.97, R² = 0.066, *p* = 0.475). Together, these findings indicate that nutritional variability in this panel is driven by accession-level differences rather than geographic provenance, and that diverse nutritional profiles are recoverable from each state sampled.

*L. siceraria* seed flour is a versatile ingredient for functional food production. C1 accessions, with the highest mineral concentrations, present a possible nutritional solution to regional dietary deficiencies in southwestern Nigeria, where bottle gourd is already culturally established. Hunter et al. [55] demonstrated that NUS can diversify diets while addressing micronutrient deficiencies. Sarfo et al. [56] showed that adding wild plant species reduced diet costs while meeting iron and zinc gaps in northern Kenya. Proietti et al. [57] demonstrated that including five NUS in Kenyan school meals met the 30% threshold for energy and essential nutrients. Onawo and Egboduku [1] observed that NUS contain high levels of carotenoids and minerals across the African diet. The present panel was sampled from four southwestern Nigerian states, and northern Nigerian and Sahelian accessions may extend the trait ranges reported here. Multi-environment trials would further strengthen stability estimates for top-ranking accessions.

## Supplementary Information

Additional file 1.

Additional file 2.

Additional file 3: Table S1. One-way analysis of variance of sixteen proximate, mineral and antioxidant traits across four states of origin (Oyo, Ogun, Osun, Ondo).

## Data Availability

All data generated or analysed during this study are included in this published article and its supplementary information files. The raw datasets used to compute the descriptive statistics, correlation matrix and multivariate analyses are all included in the supplementary files.
