# Supplementary material for: Nutritional diversity and geographic structure in 45 Lagenaria siceraria accessions from southwestern Nigeria, with comparative positioning among related cucurbit seeds
Source: BMC Plant Biol. 2026 Jul 17;26:1758. doi: 10.1186/s12870-026-09540-4 (PMC13660082; doi:10.1186/s12870-026-09540-4)
Supplement: Supplementary file 1 [file 12870_2026_9540_MOESM1_ESM.docx]

**Supplementary materials - figures, tables, R script and raw-data description**

Manuscript: Nutritional diversity and geographic structure in 45 Lagenaria siceraria accessions from southwestern Nigeria, with comparative positioning among related cucurbit seeds.

Submitted to BMC Plant Biology. Submission ID: b3438e32-02ec-474c-a852-7aa24219cf1a. This file contains all supplementary materials supporting the revised manuscript.

**Contents**

- Section 1 — Supplementary Figures (Fig. S1, Fig. S2, Fig. S3)
- Section 2 — Supplementary Tables (Table S1, Table S2, Table S3)
- Section 3 — Full R analysis script
- Section 4 — Raw data file (see separate Additional file 3)

**Section 1 — Supplementary Figures**

Figure S1. Sensitivity PCA after removal of collinear minerals

**Fig. S1.** Principal component biplot of 45 Lagenaria siceraria accessions after removal of phosphorus, calcium and iron from the trait matrix (potassium retained as the mineral representative).

PC1 (35.9%) and PC2 (23.5%) jointly explain 59.4% of the total variance, essentially identical to the 59.4% explained by the full-trait PCA (PC1 = 38.5%, PC2 = 20.8%; main-text Fig. 4). The three-cluster solution and the relative positions of accessions in the score space are preserved, confirming that the multivariate signal reflects coordinated variation across the proximate, mineral and antioxidant trait sets rather than collinearity within the mineral block alone. Ellipses are 95% confidence ellipses around the centroid of each cluster (C1 = mineral- and antioxidant-rich; C2 = tannin- and carbohydrate-rich; C3 = protein- and fat-dense).

Figure S2. Silhouette plot for the three-cluster solution

**Fig. S2**. Silhouette plot for the k = 3 partition of 45 Lagenaria siceraria accessions. Bars show silhouette width for each accession, grouped and coloured by cluster.

The mean silhouette width for the three-cluster solution is 0.312 (compared with 0.285 for k = 2, 0.329 for k = 4, and 0.302 for k = 5; see Table S3). Although the silhouette maximum occurred at k = 4, the three-cluster solution was retained on biological-interpretability grounds because the additional split at k = 4 occurred within the mineral-rich C1 group without generating a fourth functionally distinct profile.

**Figure S3. Cluster profile box plots**

Fig. S3. Distribution of trait values across the three nutritional clusters of 45 Lagenaria siceraria accessions. Box plots show the median, interquartile range, and the full range of accession-level values within each cluster, with jittered individual-accession points overlaid. Panel A — proximate composition (crude protein, moisture, crude fat, ash, crude fibre, carbohydrate). Panel B — minerals (sodium, potassium, phosphorus, calcium, iron). Panel C — phytochemicals and antioxidant activity (total phenolic content, total flavonoid content, total antioxidant capacity, tannin, DPPH scavenging).

Panel A — Proximate composition

Panel B — Minerals

Panel C — Phytochemicals and antioxidant activity

Section 2 — Supplementary Tables

Table S1. One-way ANOVA by state of origin

Table S1. One-way analysis of variance comparing sixteen proximate, mineral and antioxidant traits across the four states of origin (Oyo, Ogun, Osun, Ondo) for 45 Lagenaria siceraria accessions. F values, p values and significance codes are shown. The non-significant result for every trait at α = 0.05 indicates that nutritional and antioxidant variability in the panel is not structured by state of origin, confirming the same conclusion as the multivariate PERMANOVA test (F = 0.97, R² = 0.066, p = 0.475). Significance codes: *** p < 0.001; ** p < 0.01; * p < 0.05; ns, not significant at α = 0.05.

| Trait | F value | p value | Significance |
| --- | --- | --- | --- |
| Crude protein | 0.76 | 0.523 | ns |
| Moisture | 1.005 | 0.400 | ns |
| Crude fat | 0.362 | 0.780 | ns |
| Ash | 0.098 | 0.961 | ns |
| Crude fibre | 2.792 | 0.052 | ns |
| Carbohydrate | 0.124 | 0.945 | ns |
| Sodium | 1.427 | 0.249 | ns |
| Potassium | 1.117 | 0.353 | ns |
| Phosphorus | 1.181 | 0.329 | ns |
| Calcium | 0.736 | 0.537 | ns |
| Iron | 1.180 | 0.329 | ns |
| Total phenolic | 1.845 | 0.154 | ns |
| Flavonoid | 1.153 | 0.339 | ns |
| Total antioxidant capacity | 0.480 | 0.698 | ns |
| Tannin | 0.650 | 0.588 | ns |
| DPPH | 0.930 | 0.435 | ns |

Table S2. Two-factor ANOVA across accessions and years

Table S2. Two-factor analysis of variance for each of sixteen proximate, mineral and antioxidant traits, with Accession, Year and Accession × Year as factors and the three analytical replicates of the pooled field sample as the residual error term. Accession effects are highly significant for every trait (p < 10⁻⁵⁵), confirming substantial accession-level differentiation. Year and Accession × Year terms are non-significant for every trait, indicating that trait values are stable across the two growing seasons. Df = degrees of freedom; signif codes: *** p < 0.001; ns, not significant at α = 0.05.

| Trait | Source | Df | F value | p value | Sig |
| --- | --- | --- | --- | --- | --- |
| Crude protein | Accession | 44 | 59.38 | 3.97e-86 | *** |
| Crude protein | Year | 1 | 0.02 | 0.885 | ns |
| Crude protein | Accession × Year | 44 | 0.08 | 1 | ns |
| Crude protein | Residuals | 180 | — | — | — |
| Moisture | Accession | 44 | 23.74 | 9.89e-55 | *** |
| Moisture | Year | 1 | 0.26 | 0.613 | ns |
| Moisture | Accession × Year | 44 | 0.15 | 1 | ns |
| Moisture | Residuals | 180 | — | — | — |
| Crude fat | Accession | 44 | 214.1 | 5.8e-134 | *** |
| Crude fat | Year | 1 | 0 | 0.982 | ns |
| Crude fat | Accession × Year | 44 | 0.05 | 1 | ns |
| Crude fat | Residuals | 180 | — | — | — |
| Ash | Accession | 44 | 69.46 | 8.3e-92 | *** |
| Ash | Year | 1 | 0.07 | 0.796 | ns |
| Ash | Accession × Year | 44 | 0.04 | 1 | ns |
| Ash | Residuals | 180 | — | — | — |
| Crude fibre | Accession | 44 | 106.83 | 1.1e-107 | *** |
| Crude fibre | Year | 1 | 0 | 0.957 | ns |
| Crude fibre | Accession × Year | 44 | 0.08 | 1 | ns |
| Crude fibre | Residuals | 180 | — | — | — |
| Carbohydrate | Accession | 44 | 210.07 | 3.08e-133 | *** |
| Carbohydrate | Year | 1 | 0.03 | 0.862 | ns |
| Carbohydrate | Accession × Year | 44 | 0.03 | 1 | ns |
| Carbohydrate | Residuals | 180 | — | — | — |
| Sodium | Accession | 44 | 998.19 | 2.01e-193 | *** |
| Sodium | Year | 1 | 0.03 | 0.871 | ns |
| Sodium | Accession × Year | 44 | 0.36 | 1 | ns |
| Sodium | Residuals | 180 | — | — | — |
| Potassium | Accession | 44 | 1734.01 | 6.34e-215 | *** |
| Potassium | Year | 1 | 0 | 0.968 | ns |
| Potassium | Accession × Year | 44 | 0.95 | 0.567 | ns |
| Potassium | Residuals | 180 | — | — | — |
| Phosphorus | Accession | 44 | 1326.6 | 1.72e-204 | *** |
| Phosphorus | Year | 1 | 0 | 0.993 | ns |
| Phosphorus | Accession × Year | 44 | 0.8 | 0.812 | ns |
| Phosphorus | Residuals | 180 | — | — | — |
| Calcium | Accession | 44 | 880.06 | 1.59e-188 | *** |
| Calcium | Year | 1 | 0.23 | 0.631 | ns |
| Calcium | Accession × Year | 44 | 0.85 | 0.727 | ns |
| Calcium | Residuals | 180 | — | — | — |
| Iron | Accession | 44 | 1491.49 | 4.7e-209 | *** |
| Iron | Year | 1 | 0.05 | 0.83 | ns |
| Iron | Accession × Year | 44 | 0.7 | 0.918 | ns |
| Iron | Residuals | 180 | — | — | — |
| Total phenolic | Accession | 44 | 288.58 | 2.13e-145 | *** |
| Total phenolic | Year | 1 | 0.11 | 0.744 | ns |
| Total phenolic | Accession × Year | 44 | 0.15 | 1 | ns |
| Total phenolic | Residuals | 180 | — | — | — |
| Flavonoid | Accession | 44 | 4446.38 | 1.16e-251 | *** |
| Flavonoid | Year | 1 | 0 | 0.979 | ns |
| Flavonoid | Accession × Year | 44 | 0.33 | 1 | ns |
| Flavonoid | Residuals | 180 | — | — | — |
| Total antioxidant capacity | Accession | 44 | 3858.47 | 3.99e-246 | *** |
| Total antioxidant capacity | Year | 1 | 0.02 | 0.898 | ns |
| Total antioxidant capacity | Accession × Year | 44 | 0.11 | 1 | ns |
| Total antioxidant capacity | Residuals | 180 | — | — | — |
| Tannin | Accession | 44 | 650.78 | 8.31e-177 | *** |
| Tannin | Year | 1 | 0 | 0.947 | ns |
| Tannin | Accession × Year | 44 | 0.04 | 1 | ns |
| Tannin | Residuals | 180 | — | — | — |
| DPPH | Accession | 44 | 113.13 | 7.94e-110 | *** |
| DPPH | Year | 1 | 0.01 | 0.94 | ns |
| DPPH | Accession × Year | 44 | 0.01 | 1 | ns |
| DPPH | Residuals | 180 | — | — | — |

Table S3. Silhouette widths for k = 2 to k = 5

Table S3. Mean silhouette widths for cluster solutions ranging from k = 2 to k = 5, computed on Euclidean distances of the standardised trait matrix (sixteen proximate, mineral and antioxidant traits) with Ward's D2 linkage. The k column indicates the number of clusters; the mean silhouette column gives the average silhouette width across all 45 accessions; the n_negative column gives the number of accessions with negative silhouette width (poorly assigned to their cluster). The silhouette maximum occurred at k = 4, but the three-cluster solution was retained on biological-interpretability grounds (see Methods and Section 3.5 of the main manuscript).

| k | Mean silhouette width | Number of negative silhouettes |
| --- | --- | --- |
| 2 | 0.285 | 1 |
| 3 | 0.312 | 1 |
| 4 | 0.329 (maximum) | 1 |
| 5 | 0.302 | 2 |

Section 3 — Full R analysis script

The complete R analysis script used to produce all results, figures and tables in the revised manuscript and in this supplementary file is reproduced below. The script reads the raw data file (nutritional_long_format.xlsx, provided as Additional file 3), runs the two-factor ANOVA, PERMANOVA, PCA (full and reduced-sensitivity), hierarchical clustering with silhouette comparison, and the dietary-contribution calculations. R version 4.x is required, with packages tidyverse, readxl, vegan, cluster, factoextra, FactoMineR, Hmisc, corrplot and RColorBrewer installed.

\# ---- 1. PACKAGES -----------------------------------------------------------

pkgs <- c("tidyverse", "readxl", "vegan", "cluster", "factoextra",

"FactoMineR", "corrplot", "Hmisc", "RColorBrewer",

"psych", "ggpubr")

new <- pkgs[!pkgs %in% installed.packages()[, "Package"]]

if (length(new)) install.packages(new, dependencies = TRUE)

invisible(lapply(pkgs, library, character.only = TRUE))

\# ---- 2. READ DATA ----------------------------------------------------------

\# CHANGE THIS PATH to match your machine

file_path <- "C:/Users/user/Downloads/nutritional_long_format.xlsx"

y1 <- read_excel(file_path, sheet = "Sheet1") %>% mutate(Year = "Y1")

y2 <- read_excel(file_path, sheet = "Sheet2") %>% mutate(Year = "Y2")

dat <- bind_rows(y1, y2) %>%

rename(CRUDE_FIBRE	= `CRUDE FIBRE`,

TOTAL_PHENOLIC = `TOTAL PHENOLIC`) %>%

mutate(Accession_ID = factor(Accession_ID),

Year	= factor(Year),

Replicate	= factor(Replicate),

State	= factor(c(OD = "Ondo", OG = "Ogun",

OS = "Osun", OY = "Oyo")[substr(as.character(Accession_ID), 1, 2)],

levels = c("Ondo", "Ogun", "Osun", "Oyo")))

traits <- c("PROTEIN","MOISTURE","FAT","ASH","CRUDE_FIBRE","CHO",

"SODIUM","POTASSIUM","PHOSPHORUS","CALCIUM","IRON",

"TOTAL_PHENOLIC","FLAVONOID","TAC","TANNIN","DPPH")

cat("\n========== DATA OVERVIEW ==========\n")

cat("Total rows:", nrow(dat), "| Accessions:", nlevels(dat$Accession_ID),

"| Years:", nlevels(dat$Year), "| States:", nlevels(dat$State), "\n")

print(table(State = dat$State, Year = dat$Year))

\# ---- 3. DESCRIPTIVE STATISTICS (per-trait, pooled across years & reps) -----

cat("\n\n========== DESCRIPTIVE STATISTICS (n = 270 per trait) ==========\n")

desc <- dat %>%

pivot_longer(all_of(traits), names_to = "Trait", values_to = "v") %>%

group_by(Trait) %>%

summarise(N	= sum(!is.na(v)),

Mean	= round(mean(v, na.rm = TRUE), 3),

SE	= round(sd(v, na.rm = TRUE) / sqrt(sum(!is.na(v))), 3),

SD	= round(sd(v, na.rm = TRUE), 3),

Min	= round(min(v, na.rm = TRUE), 3),

Max	= round(max(v, na.rm = TRUE), 3),

CV_pct = round(sd(v, na.rm = TRUE) / mean(v, na.rm = TRUE) * 100, 2),

.groups = "drop")

print(as.data.frame(desc))

write.csv(desc, "01_descriptive_statistics.csv", row.names = FALSE)

\# ---- 4. TWO-FACTOR ANOVA per trait -----------------------------------------

\# Model: value ~ Accession + Year + Accession:Year

\# Lab triplicates of pooled field sample = residual error term

cat("\n\n========== TWO-FACTOR ANOVA per trait ==========\n")

cat("Model: trait ~ Accession + Year + Accession:Year\n")

cat("(lab triplicates of pooled field sample = residual error term)\n\n")

anova_out <- data.frame()

for (tr in traits) {

f <- as.formula(paste0("`", tr, "` ~ Accession_ID + Year + Accession_ID:Year"))

m <- aov(f, data = dat)

a <- as.data.frame(summary(m)[[1]])

a$Term <- rownames(a); rownames(a) <- NULL

a$Trait <- tr

anova_out <- rbind(anova_out, a)

}

anova_out <- anova_out %>%

filter(Term != "Residuals") %>%

mutate(Term	= gsub("Accession_ID:Year", "Acc:Year", Term),

Term	= gsub("Accession_ID", "Acc", Term),

F_value = round(`F value`, 2),

p_value = signif(`Pr(>F)`, 3),

signif	= ifelse(`Pr(>F)` < 0.001, "***",

ifelse(`Pr(>F)` < 0.01, "**",

ifelse(`Pr(>F)` < 0.05, "*", "ns")))) %>%

select(Trait, Term, Df, F_value, p_value, signif)

print(anova_out, row.names = FALSE)

write.csv(anova_out, "02_anova_two_factor.csv", row.names = FALSE)

\# ---- 5. POOLED ACCESSION MEANS (across years and reps) --------------------

\# Per user decision: pooled Y1+Y2 means used for PCA, clustering, correlation

acc_means <- dat %>%

group_by(Accession_ID, State) %>%

summarise(across(all_of(traits), \(x) mean(x, na.rm = TRUE)),

.groups = "drop") %>%

as.data.frame()

rownames(acc_means) <- acc_means$Accession_ID

acc_mat <- as.matrix(acc_means[, traits])

write.csv(acc_means, "03_accession_means_pooled.csv", row.names = FALSE)

\# ---- 6. PERMANOVA on State (multivariate geographic test) -----------------

cat("\n\n========== PERMANOVA: traits ~ State (9999 permutations) ==========\n")

set.seed(42)

perm <- adonis2(scale(acc_mat) ~ State, data = acc_means,

method = "euclidean", permutations = 9999)

print(perm)

\# PERMDISP — dispersion check (assumption test)

cat("\n========== PERMDISP — homogeneity of dispersion by State ==========\n")

disp <- betadisper(dist(scale(acc_mat)), acc_means$State)

print(anova(disp))

\# ---- 7. PEARSON CORRELATION with significance threshold -------------------

\# Critical |r| at α = 0.05 with n = 45 is 0.294

cat("\n\n========== PEARSON CORRELATION (n = 45 accessions) ==========\n")

cat("Significance threshold: |r| > 0.294 at α = 0.05\n\n")

rc <- rcorr(acc_mat, type = "pearson")

print(round(rc$r, 3))

write.csv(round(rc$r, 3), "04_correlation_matrix.csv")

write.csv(round(rc$P, 4), "04_correlation_pvalues.csv")

\# Correlation plot — non-significant cells blanked

png("Fig2_correlation_significance.png", width = 2400, height = 2400, res = 300)

corrplot(rc$r, method = "color", type = "upper",

p.mat = rc$P, sig.level = 0.05, insig = "blank",

tl.col = "black", tl.cex = 0.85, tl.srt = 45,

addCoef.col = "black", number.cex = 0.6,

col = colorRampPalette(c("#2166AC", "white", "#B2182B"))(200),

mar = c(0, 0, 2, 0),

title = "Pearson correlation (blank cells: p >= 0.05; n=45, critical |r|=0.294)")

dev.off()

\# ---- 8. PCA: FULL vs REDUCED (sensitivity for mineral collinearity) -------

cat("\n\n========== PCA: FULL vs REDUCED ==========\n")

pca_full <- PCA(acc_mat, scale.unit = TRUE, graph = FALSE)

cat("\n-- FULL PCA (16 traits) --\n")

print(round(get_eigenvalue(pca_full), 3))

\# Identify high-correlation pairs and drop the second variable in each

cm <- abs(cor(acc_mat))

diag(cm) <- 0

high <- which(cm > 0.85, arr.ind = TRUE)

high <- high[high[, 1] < high[, 2], , drop = FALSE]

cat("\n-- High-correlation pairs (|r| > 0.85) --\n")

pca_red <- NULL

if (nrow(high) > 0) {

for (i in seq_len(nrow(high))) {

cat(sprintf(" %-18s ~ %-18s r = %.3f\n",

colnames(acc_mat)[high[i, 1]],

colnames(acc_mat)[high[i, 2]],

cor(acc_mat)[high[i, 1], high[i, 2]]))

}

drops <- unique(colnames(acc_mat)[high[, 2]])

cat("\nDropping for reduced PCA:", paste(drops, collapse = ", "), "\n")

keep <- setdiff(colnames(acc_mat), drops)

pca_red <- PCA(acc_mat[, keep], scale.unit = TRUE, graph = FALSE)

cat("\n-- REDUCED PCA (", length(keep), "traits) --\n")

print(round(get_eigenvalue(pca_red), 3))

} else {

cat("(none)\n")

}

\# ---- 9. HIERARCHICAL CLUSTERING + SILHOUETTE COMPARISON -------------------

\# Per user decision: option B — report silhouette for k = 2..5;

\# select k = 3 based on biological interpretability.

cat("\n\n========== SILHOUETTE COMPARISON (k = 2..5) ==========\n")

sc <- scale(acc_mat)

d <- dist(sc, method = "euclidean")

hc <- hclust(d, method = "ward.D2")

sil_tbl <- data.frame()

for (k in 2:5) {

cl <- cutree(hc, k = k)

s <- silhouette(cl, d)

sil_tbl <- rbind(sil_tbl,

data.frame(k = k,

mean_silhouette = round(mean(s[, 3]), 4),

n_negative	= sum(s[, 3] < 0)))

}

print(sil_tbl)

write.csv(sil_tbl, "05_silhouette_widths.csv", row.names = FALSE)

cat("\nNote: k = 3 selected based on biological interpretability\n")

cat("(three end-use targets: mineral-rich / tannin-rich / energy-dense).\n")

\# Silhouette plot for k = 3

png("Fig_silhouette_k3.png", width = 2000, height = 1600, res = 300)

plot(silhouette(cutree(hc, k = 3), d),

border = NA, col = c("#1f77b4", "#ff7f0e", "#2ca02c"),

main = "Silhouette plot, k = 3 (Ward.D2 linkage on standardized traits)")

dev.off()

\# Assign clusters (k = 3) to accessions

acc_means$Cluster <- factor(cutree(hc, k = 3),

labels = paste0("C", 1:3))

cat("\n========== CLUSTER MEMBERSHIP ==========\n")

print(table(Cluster = acc_means$Cluster, State = acc_means$State))

cat("\n-- Accessions per cluster --\n")

for (cl in levels(acc_means$Cluster)) {

cat(cl, ":", paste(acc_means$Accession_ID[acc_means$Cluster == cl],

collapse = ", "), "\n")

}

write.csv(acc_means, "06_accession_means_with_clusters.csv", row.names = FALSE)

\# Dendrogram

png("Fig5_dendrogram.png", width = 2800, height = 1800, res = 300)

plot(hc, hang = -1, cex = 0.7,

main = "Ward.D2 hierarchical clustering of 45 L. siceraria accessions",

xlab = "", sub = "", ylab = "Euclidean distance")

rect.hclust(hc, k = 3, border = c("#1f77b4", "#ff7f0e", "#2ca02c"))

dev.off()

\# ---- 10. PCA PLOTS ---------------------------------------------------------

p_scree <- fviz_eig(pca_full, addlabels = TRUE,

main = "Scree plot — variance explained (full PCA)") +

theme_bw(base_size = 11)

ggsave("Fig_scree.png", p_scree, width = 7, height = 5, dpi = 300)

p_state <- fviz_pca_ind(pca_full, repel = TRUE,

habillage = acc_means$State,

addEllipses = TRUE, ellipse.level = 0.95,

palette = c("#E69F00","#56B4E9","#009E73","#CC79A7"),

title = "PCA — accessions coloured by state of origin") +

theme_bw(base_size = 11)

ggsave("Fig3_pca_by_state.png", p_state, width = 9, height = 7, dpi = 300)

p_cluster <- fviz_pca_ind(pca_full, repel = TRUE,

habillage = acc_means$Cluster,

addEllipses = TRUE, ellipse.level = 0.95,

palette = c("#1f77b4","#ff7f0e","#2ca02c"),

title = "PCA — accessions coloured by cluster (C1/C2/C3)") +

theme_bw(base_size = 11)

ggsave("Fig4_pca_by_cluster.png", p_cluster, width = 9, height = 7, dpi = 300)

p_load <- fviz_pca_var(pca_full, repel = TRUE, col.var = "contrib",

gradient.cols = c("#00AFBB","#E7B800","#FC4E07"),

title = "PCA — variable loadings (full)") +

theme_bw(base_size = 11)

ggsave("Fig_pca_loadings.png", p_load, width = 9, height = 7, dpi = 300)

if (!is.null(pca_red)) {

p_red <- fviz_pca_ind(pca_red, repel = TRUE,

habillage = acc_means$Cluster,

addEllipses = TRUE, ellipse.level = 0.95,

palette = c("#1f77b4","#ff7f0e","#2ca02c"),

title = "PCA — reduced (collinear minerals removed)") +

theme_bw(base_size = 11)

ggsave("FigS_pca_reduced_sensitivity.png", p_red,

width = 9, height = 7, dpi = 300)

}

\# ---- 11. CLUSTER PROFILE BOXPLOTS (R3 #10) ---------------------------------

proximate <- c("PROTEIN","MOISTURE","FAT","ASH","CRUDE_FIBRE","CHO")

minerals <- c("SODIUM","POTASSIUM","PHOSPHORUS","CALCIUM","IRON")

phytos	<- c("TOTAL_PHENOLIC","FLAVONOID","TAC","TANNIN","DPPH")

cluster_long <- acc_means %>%

pivot_longer(all_of(traits), names_to = "Trait", values_to = "Value")

make_box <- function(tr_set, ttl) {

ggplot(cluster_long %>% filter(Trait %in% tr_set),

aes(x = Cluster, y = Value, fill = Cluster)) +

geom_boxplot(alpha = 0.85, outlier.shape = NA) +

geom_jitter(width = 0.18, size = 1.3, alpha = 0.55) +

facet_wrap(~ Trait, scales = "free_y", nrow = 1) +

scale_fill_manual(values = c("#1f77b4","#ff7f0e","#2ca02c")) +

labs(title = ttl, x = NULL, y = NULL) +

theme_bw(base_size = 11) +

theme(legend.position = "none",

strip.background = element_rect(fill = "grey92"))

}

ggsave("Fig_cluster_proximate.png", make_box(proximate, "Proximate composition by cluster"),

width = 13, height = 4.5, dpi = 300)

ggsave("Fig_cluster_minerals.png", make_box(minerals, "Mineral composition by cluster"),

width = 12, height = 4.5, dpi = 300)

ggsave("Fig_cluster_phytos.png", make_box(phytos, "Phytochemicals & antioxidant activity by cluster"),

width = 12, height = 4.5, dpi = 300)

\# ---- 12. CLUSTER MEANS ± SE (R1 minor #6) ----------------------------------

cluster_meanSE <- cluster_long %>%

group_by(Cluster, Trait) %>%

summarise(N	= n(),

Mean = round(mean(Value, na.rm = TRUE), 3),

SE	= round(sd(Value, na.rm = TRUE) / sqrt(n()), 3),

.groups = "drop") %>%

mutate(Mean_SE = paste0(Mean, " ± ", SE)) %>%

select(Cluster, Trait, Mean_SE) %>%

pivot_wider(names_from = Cluster, values_from = Mean_SE)

cat("\n\n========== CLUSTER MEANS ± SE (revised Table 2) ==========\n")

print(as.data.frame(cluster_meanSE))

write.csv(cluster_meanSE, "07_cluster_means_SE.csv", row.names = FALSE)

\# ---- 13. FIGURE 6: RDA CONTRIBUTION BAR CHART -------------------------------

\# Reference values: FAO/WHO 2004 (Ca, Fe), IOM 1997 (P), WHO 2012 (K, Na),

\# FAO/WHO/UNU 2007 (Protein). Adult reference, in mg/day.

rda <- data.frame(

Trait = c("PROTEIN","FAT","CRUDE_FIBRE",

"SODIUM","POTASSIUM","PHOSPHORUS","CALCIUM","IRON"),

RDA_mg = c(50000, 70000, 25000,

2000, 3510, 700, 1000, 13.7),

Source = c("FAO/WHO/UNU 2007", "IOM 2005", "WHO 2003",

"WHO 2012", "WHO 2012", "IOM 1997", "FAO/WHO 2004", "FAO/WHO 2004"))

cat("\n\n========== RDA REFERENCE VALUES USED ==========\n")

print(rda)

write.csv(rda, "08_rda_references.csv", row.names = FALSE)

\# Per-accession % RDA from 100 g seed flour

contrib <- acc_means %>%

select(Accession_ID, Cluster, all_of(rda$Trait)) %>%

pivot_longer(-c(Accession_ID, Cluster),

names_to = "Trait", values_to = "Value") %>%

left_join(rda, by = "Trait") %>%

mutate(Value_mg = ifelse(Trait %in% c("PROTEIN","FAT","CRUDE_FIBRE"),

Value * 1000, Value),

Pct_RDA = Value_mg / RDA_mg * 100)

\# Cluster mean ± SE % RDA

contrib_cluster <- contrib %>%

group_by(Cluster, Trait) %>%

summarise(Mean_pct = mean(Pct_RDA, na.rm = TRUE),

SE_pct	= sd(Pct_RDA, na.rm = TRUE) / sqrt(n()),

.groups = "drop")

contrib_cluster$Trait <- factor(contrib_cluster$Trait,

levels = c("PROTEIN","FAT","CRUDE_FIBRE",

"PHOSPHORUS","POTASSIUM","CALCIUM",

"IRON","SODIUM"))

p_rda <- ggplot(contrib_cluster,

aes(x = Trait, y = Mean_pct, fill = Cluster)) +

geom_col(position = position_dodge(0.8), width = 0.72) +

geom_errorbar(aes(ymin = pmax(Mean_pct - SE_pct, 0),

ymax = Mean_pct + SE_pct),

position = position_dodge(0.8), width = 0.22) +

geom_hline(yintercept = 100, linetype = "dashed", colour = "red", linewidth = 0.5) +

scale_fill_manual(values = c("#1f77b4","#ff7f0e","#2ca02c")) +

labs(title = "Figure 6. Contribution of 100 g L. siceraria seed flour to adult daily reference intakes",

subtitle = "Dashed line = 100% of reference intake. Bars are cluster means +/- SE.",

y = "% of reference daily intake", x = NULL, fill = "Cluster") +

theme_bw(base_size = 11) +

theme(axis.text.x = element_text(angle = 30, hjust = 1),

legend.position = "top",

plot.title = element_text(size = 11, face = "bold"))

ggsave("Fig6_RDA_contribution.png", p_rda,

width = 10, height = 6, dpi = 300)

print(p_rda)

write.csv(contrib_cluster, "09_RDA_contribution_per_cluster.csv", row.names = FALSE)

contrib_wide <- contrib %>%

select(Accession_ID, Cluster, Trait, Pct_RDA) %>%

mutate(Pct_RDA = round(Pct_RDA, 1)) %>%

pivot_wider(names_from = Trait, values_from = Pct_RDA)

write.csv(contrib_wide, "09b_RDA_per_accession.csv", row.names = FALSE)

\# ---- 14. TOP-RANKING ACCESSIONS PER TRAIT (revised Table 3) ----------------

cat("\n\n========== TOP-3 ACCESSIONS PER TRAIT (revised Table 3) ==========\n")

top3 <- list()

for (tr in traits) {

ord <- order(acc_means[[tr]], decreasing = TRUE)[1:3]

top3[[tr]] <- data.frame(Trait	= tr,

Rank	= 1:3,

Accession = as.character(acc_means$Accession_ID[ord]),

State	= as.character(acc_means$State[ord]),

Value	= round(acc_means[[tr]][ord], 3),

Cluster = as.character(acc_means$Cluster[ord]))

}

top3_tbl <- do.call(rbind, top3)

print(top3_tbl, row.names = FALSE)

write.csv(top3_tbl, "10_top3_per_trait.csv", row.names = FALSE)

\# ---- 15. SUMMARY ----------------------------------------------------------

cat("\n\n========== ANALYSIS COMPLETE ==========\n")

cat("All CSVs and PNGs saved to working directory:", getwd(), "\n\n")

cat("CSV outputs:\n",

" 01_descriptive_statistics.csv\n",

" 02_anova_two_factor.csv\n",

" 03_accession_means_pooled.csv\n",

" 04_correlation_matrix.csv\n",

" 04_correlation_pvalues.csv\n",

" 05_silhouette_widths.csv\n",

" 06_accession_means_with_clusters.csv\n",

" 07_cluster_means_SE.csv\n",

" 08_rda_references.csv\n",

" 09_RDA_contribution_per_cluster.csv\n",

" 09b_RDA_per_accession.csv\n",

" 10_top3_per_trait.csv\n\n",

"Figure outputs (300 dpi PNG):\n",

" Fig2_correlation_significance.png\n",

" Fig3_pca_by_state.png\n",

" Fig4_pca_by_cluster.png\n",

" Fig5_dendrogram.png\n",

" Fig6_RDA_contribution.png\n",

" Fig_silhouette_k3.png\n",

" Fig_scree.png\n",

" Fig_pca_loadings.png\n",

" FigS_pca_reduced_sensitivity.png\n",

" Fig_cluster_proximate.png\n",

" Fig_cluster_minerals.png\n",

" Fig_cluster_phytos.png\n", sep = "")

Section 4 — Raw data file

The complete raw analytical dataset is provided as a separate file: nutritional_long_format.xlsx (Additional file 3). BMC Plant Biology accepts Excel files (.xlsx) for supplementary data and no conversion is required.

The file contains two sheets:

Sheet 1 — Year 1 (2023 growing season): 135 rows representing 45 accessions × 3 analytical replicates of the pooled field sample. Columns include accession code, state of origin, year, and the 16 measured traits.

Sheet 2 — Year 2 (2024 growing season): 135 rows representing the same 45 accessions × 3 analytical replicates in the second growing season.

Total: 270 measurements per trait across the panel. Accession codes follow the convention [State][Number], where the state prefix indicates the state of origin: OD = Ondo, OG = Ogun, OS = Osun, OY = Oyo.

Sixteen analytical traits in the file:

PROTEIN (% dw); MOISTURE (% fw); FAT (% dw); ASH (% dw); CRUDE_FIBRE (% dw); CHO (% dw, by difference); SODIUM (mg/100 g); POTASSIUM (mg/100 g); PHOSPHORUS (mg/100 g); CALCIUM (mg/100 g); IRON (mg/100 g); TOTAL_PHENOLIC (mg GAE/g); FLAVONOID (mg QE/g); TAC (mg TE/g); TANNIN (mg/g); DPPH (% scavenging).
