# Supplementary material for: Nutritional diversity and geographic structure in 45 Lagenaria siceraria accessions from southwestern Nigeria, with comparative positioning among related cucurbit seeds
Source: BMC Plant Biol. 2026 Jul 17;26:1758. doi: 10.1186/s12870-026-09540-4 (PMC13660082; doi:10.1186/s12870-026-09540-4)
Supplement: Supplementary file 2 [file 12870_2026_9540_MOESM3_ESM.docx]

**Supplementary file**

**Table S1.** One-way analysis of variance of sixteen proximate, mineral and antioxidant traits across four states of origin (Oyo, Ogun, Osun, Ondo).

| **Trait** | **F value** | **P value** | **Sig** |
| --- | --- | --- | --- |
| PROTEIN | 0.76 | 0.5228 | ns |
| MOISTURE | 1.005 | 0.4003 | ns |
| FAT | 0.362 | 0.7804 | ns |
| ASH | 0.098 | 0.9608 | ns |
| CRUDE_FIBRE | 2.792 | 0.0524 | ns |
| CHO | 0.124 | 0.9452 | ns |
| SODIUM | 1.427 | 0.2487 | ns |
| POTASSIUM | 1.117 | 0.3531 | ns |
| PHOSPHORUS | 1.181 | 0.3288 | ns |
| CALCIUM | 0.736 | 0.5366 | ns |
| IRON | 1.18 | 0.329 | ns |
| TOTAL_PHENOLIC | 1.845 | 0.1541 | ns |
| FLAVONOID | 1.153 | 0.3394 | ns |
| TAC | 0.48 | 0.6977 | ns |
| TANNIN | 0.65 | 0.5876 | ns |
| DPPH | 0.93 | 0.4349 | ns |
